# Performance and emission analysis of sustainable rice bran-Calophyllum-CNSL biodiesel blends in a conventional diesel engine

**DOI:** 10.1371/journal.pone.0360637

**Published:** 2026-10-30

**Authors:** Hariharan Kondin Balakrishnan, Bella Raman Senthil Kumar, Rathinavel Subbiah, Senthilkumar Thangarajan Sivasankaran, Milon Selvam Dennison

**Affiliations:** 1 Department of Mechanical Engineering, K. Ramakrishnan College of Technology, Trichy, Tamilnadu, India; 2 Department of Aeronautical Engineering, Nehru Institute of Engineering and Technology, Coimbatore, Tamilnadu, India; 3 Department of Mechanical Engineering, SRM Institute of Science and Technology, Tiruchirappalli, Tamilnadu, India; 4 Centre for Material Science, Karpagam Academy of Higher Education, Coimbatore, Tamilnadu, India; 5 Department of Mechanical Engineering, Karpagam Academy of Higher Education, Coimbatore, Tamilnadu, India; 6 Department of Mechanical Engineering, School of Engineering and Applied Sciences, Kampala International University, Ishaka-Bushenyi, Uganda; Vietnam Maritime University, VIET NAM

## Abstract

Diesel engines are often run on blended fuels to evaluate the adaptability and alternative energy source with changing engine components. The study compares the engine performance and emission characteristics of diesel engines operating on pure diesel and biodiesel blends. Tests were carried out on a single cylinder, four stroke, water cooled direct injection diesel engine at constant speed of 1500 rpm. The 10%, 20% and 30% bio-oils by volume (B10, B20 and B30) biodiesel blends were tested at different engine loads in comparison to regular diesel without any engine modifications. The diesel engine performance was evaluated by brake power (BP), brake specific fuel consumption (BSFC), total fuel consumption (TFC) and brake thermal efficiency (BTE). Also, emissions characteristics such as carbon dioxide (CO2), nitrogen oxides (NOx) and unburned hydrocarbons (UBHC) were measured. The results revealed that all the biodiesel blends had the same braking power for all the fuels with a maximum value of 4.26KW under all loads. Because biodiesel has a reduced calorific value, performances like TFC and BSFC improved with its presence; this impact was particularly noticeable for the B30 mix. Nevertheless, the B20 blend achieved a slight increase in TFC at medium and higher loads, leading to effective fuel use. Due to better combustion aided by the oxygenated fuel characteristic, B20 outperforms the other blends, obtaining a higher BTE of 30.2% than diesel at greater loads. Higher biodiesel concentration increased the UBHC emission since it had the maximum viscosity and decreased fuel volatility. All things considered, the B20 blend offers the best balance between thermal efficiency, emission characteristics, and fuel consumption.

## 1. Introduction

Cashew nut oil, with an oil content of 32%, yields about 87% biodiesel after pretreatment due to its high fatty acid value, and its fuel properties meet biodiesel standards, making it suitable for diesel engines [1]. India, being the world’s largest cashew producer, generates significant liquid of cashew nut shell (CNSL), a valuable by-product for biofuel applications [2]. Biodiesel synthesized from waste cooking oil shows favorable viscosity, cetane number, and oxidation stability, while ternary biodiesel–diesel–propanol blends improve cold-flow behavior and reduce major emissions [3]. Algae–diesel blends, particularly A20, provide higher thermal efficiency and lower CO, HC, smoke, and particulate emissions compared with diesel, although NOx tends to rise [4]. Studies on rice bran oil methyl esters report that blends such as B10 and B20 show performance similar to diesel, whereas higher blends like B40 exhibit poorer combustion and increased emissions [5]. Various conversion technologies—transesterification, pyrolysis, and micro-emulsification—enable biodiesel production from diverse oils, with transesterification being the most widely used due to better fuel quality [6]. Experiments on rice bran biodiesel with n-butanol indicate increased BSFC but reduced CO and smoke emissions, while NOx decreases with butanol addition [7]. Fig seed oil biodiesel blends, especially B10, show improved combustion profiles validated through numerical simulations [8]. CNSL biodiesel combined with 10% ethanol significantly reduces CO_2_, HC, and NO emissions while maintaining acceptable performance, confirming its suitability for diesel engines without modification [9]. Calophyllum inophyllum biodiesel (B20) performs best at higher injection pressures (210 bar), delivering lower emissions and comparable efficiency to diesel [10], while other studies confirm that biodiesel blends maintain engine thermal efficiency similar to diesel as torque increases [11]. Optimization studies using Taguchi and ANOVA methods highlight the strong influence of engine load, DEE ratio, palm oil proportion, injection timing on performance and emissions [12].

Biodiesel combustion typically produces higher NOx due to oxygen content and increased premixed combustion, although hydrogen addition can mitigate NOx and smoke [13]. Cetane improvers such as neopentane reduce NOx and smoke, with 3 mL showing optimal results [14]. Reviews indicate that biodiesel cannot fully replace diesel under extreme climates but remains viable in controlled environments despite lower heating value and increased NOx emissions [15]. Pyrolysis oils from different biomass and waste feedstocks have been studied for fuel production and combustion [16–18]. These studies show the production of alternative fuels from waste and renewable resources through thermochemical conversion pathways. Combustion modeling studies reveal that ethanol spray behavior is insensitive to droplet size, unlike pyrolysis oils, and local reaction–diffusion-based models predict flame behavior effectively [19]. Long-term engine tests suggest that blends of 60–70% PPO perform efficiently at high engine loads, though further optimization of injection parameters is required [20].

Microwave-assisted pyrolysis modeling of lignin confirms changes in electric field distribution upon introduction of biomass and validates kinetic modeling for product distribution prediction [21]. Combustion of LDPE700 produces lower NOx and CO emissions than diesel, while EVA blends show longer ignition delay and reduced thermal efficiency [22]. Integrating acid leaching with pyrolysis enhances cost-effectiveness in producing pyrolysis sugars and heating oils, particularly when using low-cost biomass wastes [23]. Thin films produced via nebulized spray pyrolysis exhibit improved electrical mobility and structural stability, indicating their suitability for PEC solar cells [24]. Finally, sewage-sludge-derived bio-oil generated through fluidized bed pyrolysis offers high energy potential comparable to heavy fuel oil, suggesting it could serve as a competitive petroleum substitute with proper upgrading [25].

Recent studies have also shown the feasibility in using complex biodiesel formulations for enhancing performance and emissions of diesel engines. The combustion features and brake thermal efficiency of biodiesel of Sterculia fetida with decanol blends have been improved with the reduction of exhaust emissions due to the enhancement of fuel atomization and availability of oxygen [26–28]. Sterculia fetida biodiesel blends with butanol also showed lower emissions and higher combustion efficiency due to better fuel-air mixing and evaporation characteristics. The addition of ignition improvers to Calophyllum inophyllum biodiesel is shown to significantly reduce the emissions and improve the combustion performance [29]. Sterculia fetida biodiesel is a promising renewable feedstock for the production of sustainable biodiesel. The results support the studies about multi-feedstock biodiesel blends and show the importance of fuel formulation and blending approaches to optimize the performance of diesel engines fueled by biodiesel.

The oxygenated nature of biodiesel and synergistic fuel properties of rice bran oil, Calophyllum inophyllum oil and CNSL support the notion that moderate biodiesel blending ratios improve the combustion quality, thermal efficiency and reduce the harmful exhaust emissions with satisfactory engine performance. Hence, the main objective of this research work is to find out the best blend ratio for better fuel efficiency and less environmental impact. The performance and emission characteristics of ternary biodiesel blends (B10, B20 and B30) at different loads on a single cylinder direct injection diesel engine are also investigated.

This study fills the research gap by experimentally evaluating the combined effects of multi-feedstock biodiesel blends prepared from rice bran oil, Calophyllum inophyllum oil and cashew nut shell liquid (CNSL) on engine performance and exhaust emissions in unmodified engine. Unlike most of the previous researches focusing on single-source biodiesel or binary blends, this work systematically evaluates the ternary bio-oil blends (B10, B20, and B30) operated at a constant speed and varying load conditions, which facilitates a realistic assessment of the fuel adaptability in the conventional diesel engine. The study is concerned with the comparative analysis of braking power, fuel consumption characteristics, thermal efficiency and regulated emissions (UBHC, NO_x_ and CO_2_) with a view to provide a thorough understanding of combustion behavior and energy utilization. The results provide experimentally verified insights into optimum blending ratio for maximizing efficiency gains and emission trade-offs, a practical step toward deployment of sustainable biodiesel blends without engine modification.

## 2. Experimental setup

### 2.1. Heater

A burner setup in the firebox of an anaerobic condition process heater as shown in Fig 1. This setup is used for the cracking of hydrocarbons in the olefin manufacturing process. The firebox’s hearth is directly heated by the burner arrangement, turning it in to a radiant surface. The selected burner arrangement encourages amore even vertical heat flux distribution over the firebox height and raises the heat flux in the bottom portion of the firebox. The base burners can use porous ceramic burner or be made to fire horizontally across the hearth. The base burner operates in tandem woth optional wall burners placed in the upper firebox area and hearth burners that fire vertically. The hearter’s dimensions are length = 1000 mm and breath = 170 mm.

**Fig 1:**
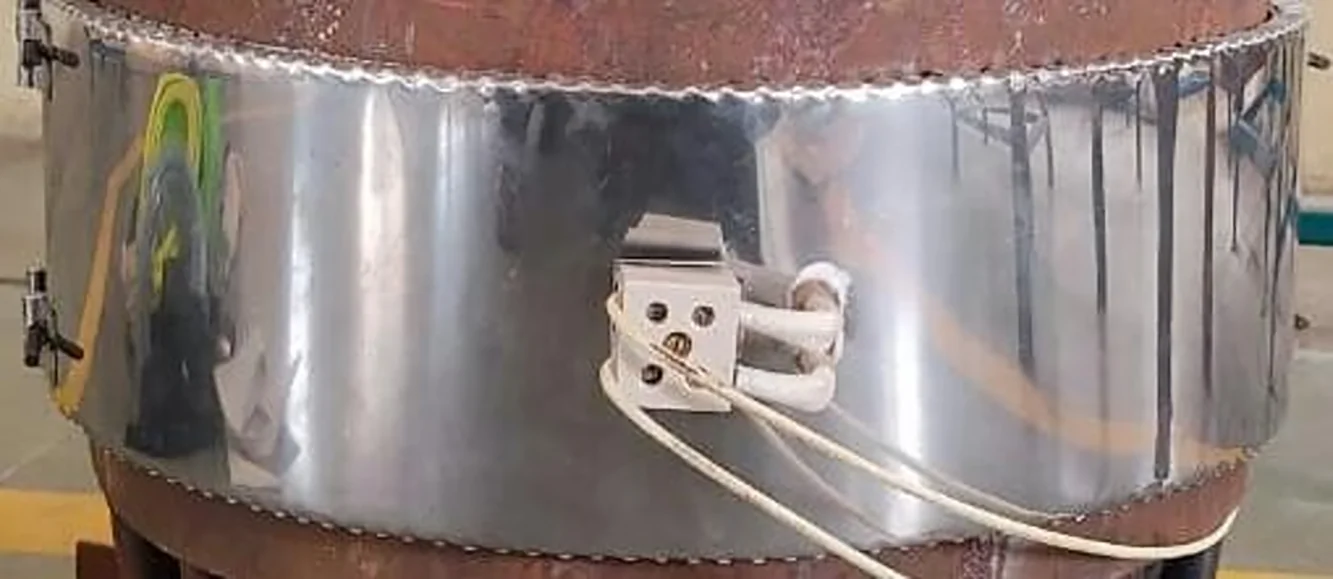
Heater.

### 2.2. Condenser

A condenser as shown in Fig 2 is a heat exchange device used in various systems to induce the phase transition of a working fluid from vapor to liquid by removing heat. The rejected thermal energy is absorbed by the coolant circulating through the condenser.

**Fig 2:**
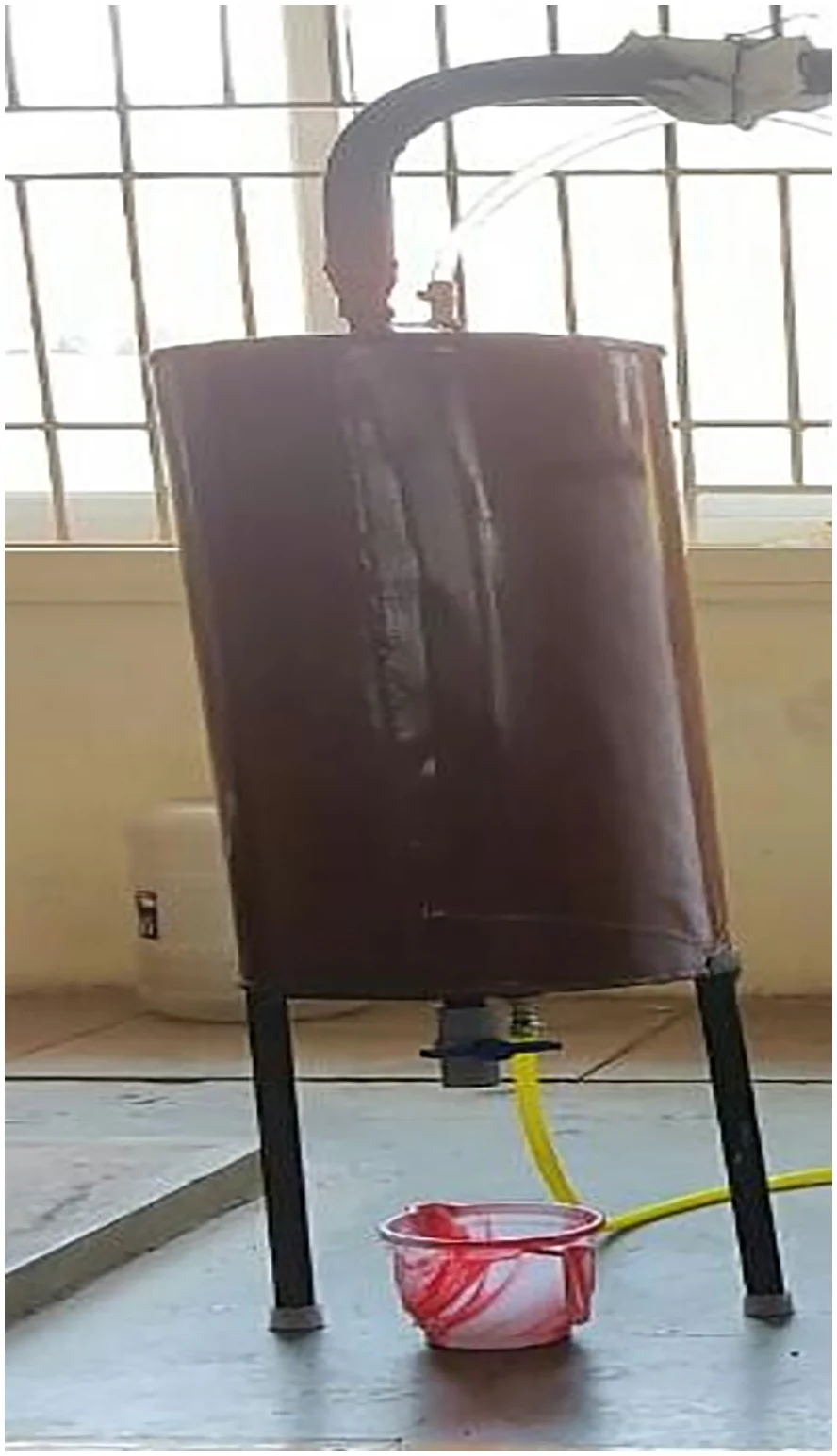
Condenser.

Condensing devices are available in multiple design forms and size ranges, extending from portable or small units to large-scale industrial condensers used in processing facilities. For instance, in a domestic refrigerator, the condenser dissipates the heat extracted from the refrigerated space to the external environment. The specifications of the condenser are Height (h) = 677 mm, Diameter = 312 mm Type = Cross flow.

### 2.3. Gas and liquid separator

Up to 99% of moisture and solid particulates larger than 10 μm can be removed using gas–liquid separation units. Such separators are typically installed after intercooling or aftercooling systems to prevent carryover of entrained liquids that may adversely affect later compression stages or downstream processes.

Gas–liquid separators are commonly employed in main air distribution lines supplying pneumatic equipment, including air chucks, air jets, and paint spraying units, to eliminate entrained moisture and particulates. They are also positioned ahead of steam turbines to prevent blade damage resulting from wet steam, pipe scale, and other erosive solids.

In steam distribution systems, separators are installed to guarantee the delivery of clean and dry steam to heat exchangers, pressure-reducing stations, temperature regulators, metering devices, and associated process equipment. The specification of gas and liquid separator is Diameter (D) = 100 mm Depth (h) = 340 mm, Volume (V) = 2.669*10−3mm3

### 2.4. Eddy current dynamometer

An absorption dynamometer known as an eddy current dynamometer a rotor disc, typically fabricated from copper or steel and coupled to the engine shaft, rotates within a stator carrying electromagnetic coils. Interaction between the rotating conductive disc and the magnetic field induces eddy currents that produce a counteracting torque, resulting in controlled loading of the engine. Load regulation is achieved by adjusting the excitation current of the electromagnets.

### 2.5. AVL Di gas analyzer

The engine’s exhaust emissions were assessed using an AVL DI gas analyzer. The analyzer measures CO_2_, HC, CO, NOx, and SOx emissions, with CO and CO_2_ quantified on a volumetric percentage basis, while HC, SOx, and NOx concentrations are recorded in ppm. Fig 3 shows the configuration of the AVL gas analyzer. Table 1 represents the specification of the AVL Di gas analyzer.

**Table 1:** Specifications of AVL DI gas analyzer.

MEASUREMENT PARAMETER	PRINCIPLE OF MEASUREMENT	RANGE	RESOLUTION
Opacity (HU)	Light Absorption	0-99.99%	0.01%
Opacity(K)	Light Absorption	0-99.99%	0.001/m
RPM	Piezo based Battery based	0-6000 0-6000	1 10
OT	RTD	0-150°C	1°C
Linearity, Repeatability, Zero and Span drift	+0.1/m		
Response Time	Physical: < 0.4sec, Electrical: < 1m sec		
Smoke Column	215 mm long (430 mm folded length)		
Warm up Time	<15 min		
Dimensions	460 mm x 145 mm x 275 mm		
Weight	8 kg		
Power supply	11-13 V DC/230 V AC + 10%		
Power	<75 W		

**Fig 3:**
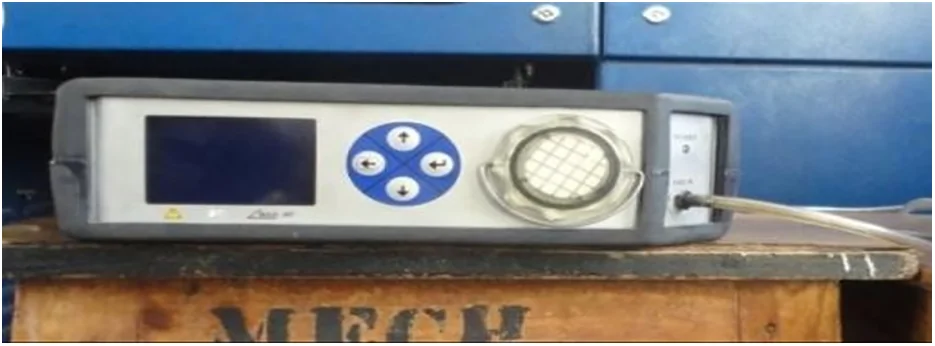
AVL DI gas analyzer.

### 2.6. Experimental procedure

#### 2.6.1. Synthesis of bio-diesel.

In this work anaerobic condition process had been used. First 200 ml methanol was mixed with 15g KOH. The KOH image is shown in Fig 4. Methanol and potassium hydroxide were combined in a glass container and agitated until complete dissolution of the catalyst was obtained, producing methoxide through an exothermic reaction. Owing to the corrosive nature of methoxide, necessary safety precautions were implemented during handling. The methoxide solution was then added to one liter of oil mixed with 5 mL of sulfuric acid and preheated to about 65 °C. The reaction was sustained at 60–61 °C with constant stirring at approximately 600 rpm for one hour. To effectively separate the glycerol from the ester phase, the reaction combination was let to relax for a full day after finishing.

**Fig 4:**
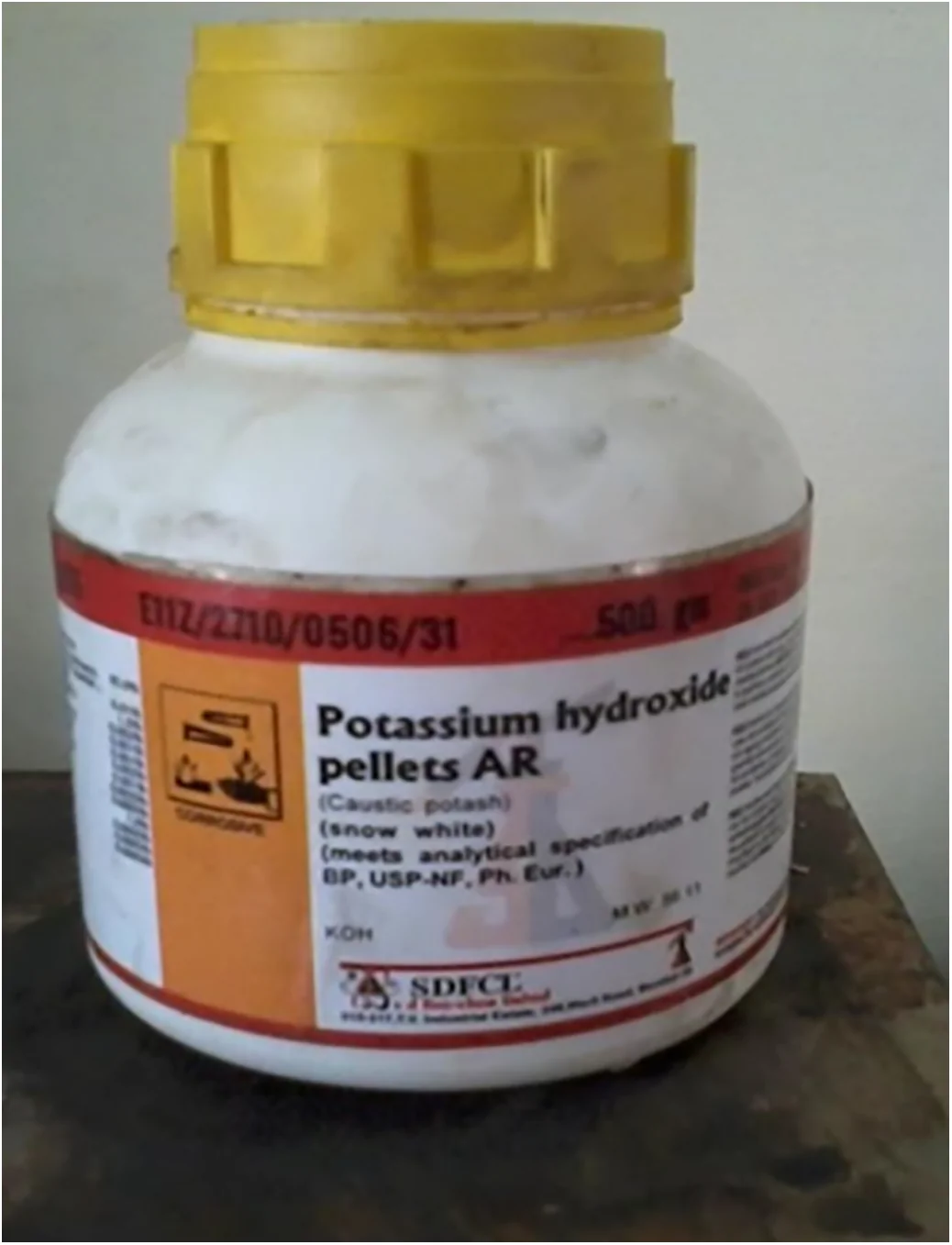
Potassium hydroxide (KOH) base catalyst.

The reaction mixture divided into two distinct layers upon settling, with glycerol accumulating as the heavier lower phase and biodiesel forming the upper phase. Separation of the glycerol was achieved by either gravity-based decantation or centrifugation. If impurities were present in the initial oil, a thin intermediate layer composed of soaps and other contaminants was observed. The produced biodiesel generally retained residual moisture and methanol, with boiling points of approximately 100 °C and 65 °C, respectively, along with trace amounts of soap. At soap concentrations between 300 and 500 ppm, methanol could be removed by vaporization and subsequently recycled into the process. At higher soap levels, methanol acts as a co-solvent, complicating its separation from the biodiesel phase. This setup for transesterification process is shown in Fig 5.

**Fig 5:**
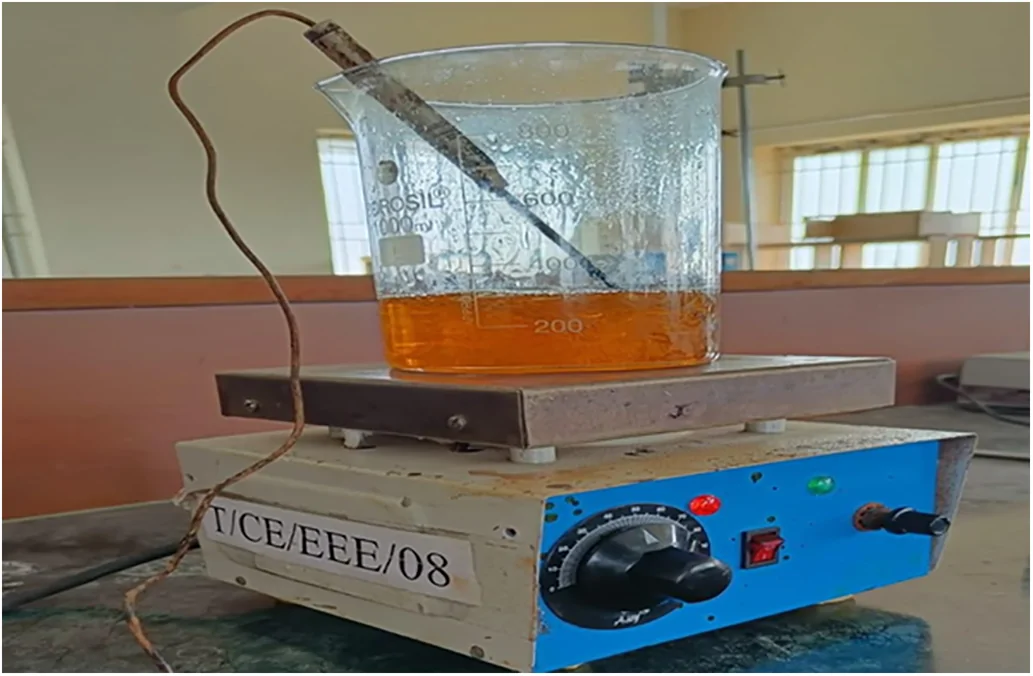
Mixture of Methanol-KOH catalyst solutions for transesterification process.

Removal of methanol leads to the precipitation of soap as a viscous sludge. Heating the biodiesel above 100 °C enables effective removal of both residual water and methanol. The drying process involved heating the biodiesel to about 105 °C for one hour, after which a clear appearance was observed, corresponding to a moisture content of less than 0.1%. The final step involved filtration, yielding a high-quality biodiesel product.

#### 2.6.2. Alcohol removal.

Through distillation process the remaining alcohol in both layers were separated after the partition of biodiesel and the glycerol phases. The distilled alcohol was recovered and reused in the process. Careful attention was required to avoid water accumulation in the recovered alcohol stream, as shown in Fig 6.

**Fig 6:**
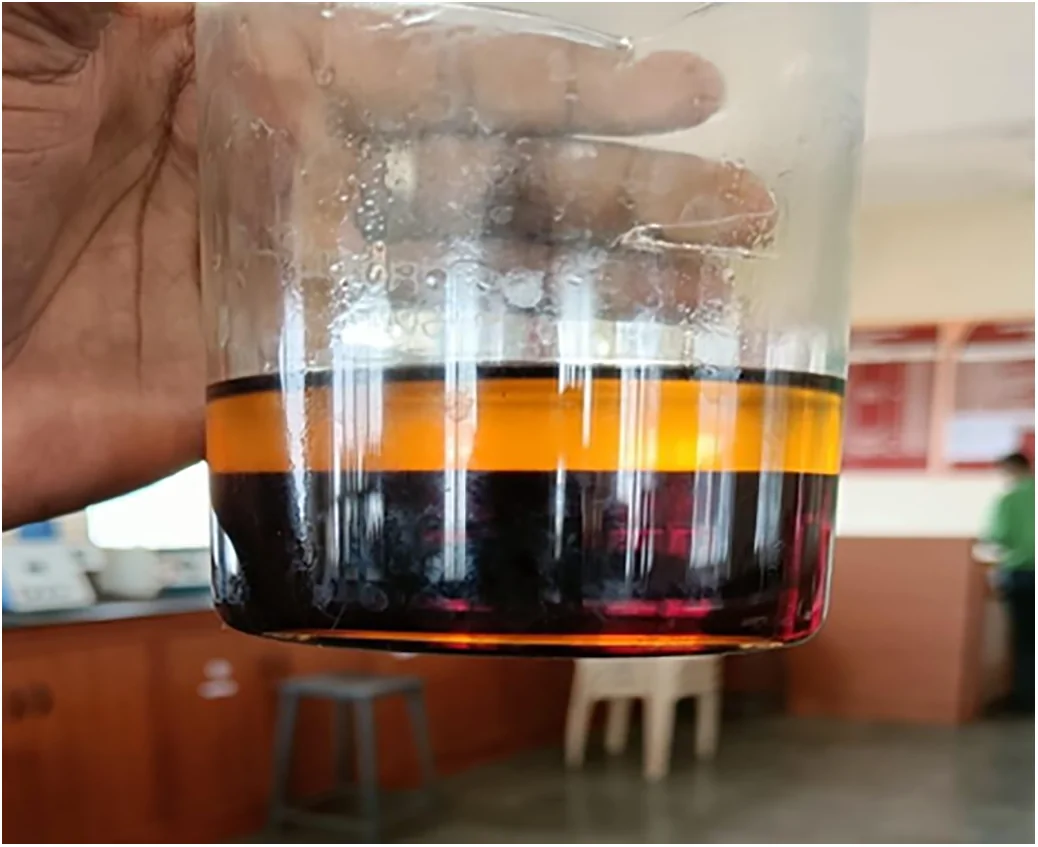
Rice bran oil after oil Transesterification.

Following glycerol separation and alcohol distillation, the crude biodiesel underwent purification through gentle warm water washing to remove traces of catalyst and soap. Air bubbling was employed during the washing process, and washing was continued until the effluent water ran clear, confirming the complete removal of impurities from the biodiesel.

Following the separation of biodiesel and glycerol, residual alcohol present in both phases was removed using a distillation process. On the other hand, the distillation equipment is utilized to recover and reuse the alcohol. It is important to make sure that the recovered alcohol stream doesn’t get too wet.

#### 2.6.3. Biodiesel drying.

The water content is removed from the biodiesel at the last stage, resulting a transparent yellowish-brown fuel with a similar viscosity of diesel. In order to produce a colorless biodiesel, the biodiesel is further decontaminated to eliminate the minor impurities.

The final stage in biodiesel production involves removing residual water, yielding a transparent amber-yellow fuel with viscosity similar to that of petroleum diesel. In some configurations, the biodiesel undergoes further distillation to eliminate minor color impurities, producing a nearly colorless product. The study of pyrolyzate shows that its calorific value is similar to diesel although its cetane number, viscosity, pour point, flash point and flash point are lower than diesel fuel. Cracking is the thermal or catalytic conversion of a molecule to different chemical entities (Fig 7).

**Fig 7:**
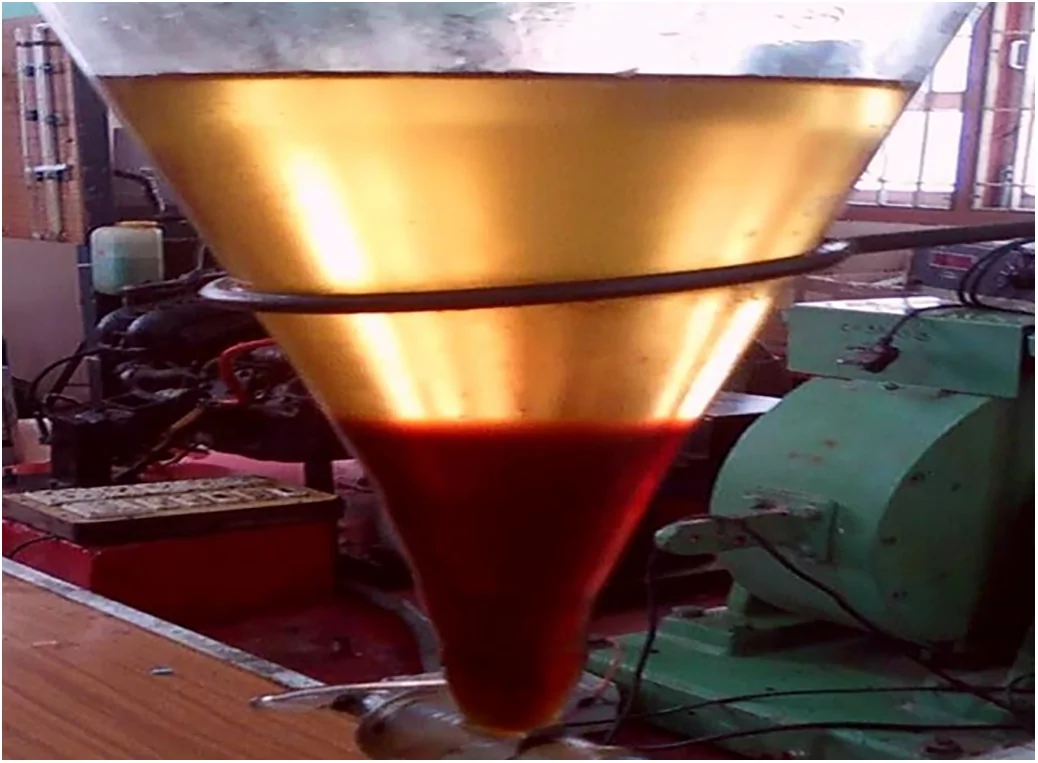
Oil extraction.

#### 2.6.4. Preparation of blends.

In this research work, the blends are prepared with mixing of existing available diesel and bio-oils to analyze the performance of the engine. The properties of the diesel and various oils are shown in the Table 2. The various combination of the blends is mentioned below.

**Table 2:** Properties of prepared blended oil.

Properties	Calophyllum Inophyllum Oil	Cashewnut Shell Oil	Ricebran Oil
Flash point, oc	224	65	76
Fire point, oc	253	70	104
Specific gravity at 15 oc	0.952	0.866	0.921
Viscosity at 40 oc, mm2/sec	4.2	4.1	4.63
Cetane number	59.5	33	52
Calorific Value (MJ/kg)	39.4	40.1	38.8

Various bends we used to get the performances tests are Diesel B10, B20, and B30.

- B10 - 900 ml of diesel +50 ml of rice bran oil + 25 ml of callophyllum inophyllum + 25 ml of cashew shell oil.
- B20 – 800 ml of diesel + 100 ml of rice bran oil + 50 ml of callophyllum inophyllum +50 ml of cashew shell oil.
- B30 - 700 ml of diesel + 150 ml of rice bran oil + 75 ml of callophyllum inophyllum +75 ml of cashew shell oil.

All experiments were performed using commercial diesel gasoline purchased from a local gas station as reference fuel. Table 3 provides the physical properties of diesel fuel. Diesel was used to satisfy IS 1460 specification and was used as a reference fuel for comparison of biodiesel blends. When operating in low temperatures with biodiesel, cold-flow properties are important.

**Table 3:** Properties of diesel fuel.

Property	Value
Fuel Type	Commercial Diesel
Standard	IS 1460/ ASTM D975
Density at 15°C	0.824 g/cm³
Kinematic Viscosity at 40°C	3.6 mm²/s
Cetane Number	52
Flash Point	55°C
Fire Point	58°C
Calorific Value	42.5 MJ/kg
Sulfur Content	< 50 ppm

The presence of long chain fatty acid esters causes the pour and cloud points of biodiesel to be higher than that of petroleum diesel. The aromatic chemicals found in biodiesel made from CNSL may positively influence the low-temperature flow behavior to a certain degree. The two feedstocks considered in this work, rice bran oil and Calophyllum inophyllum biodiesel can result in relatively poor cold-flow properties. The operation of the engine did not show any problems with cold start, gasoline gelling, or filter blockage. Only blends B10, B20 and B30 were tested under the normal ambient conditions of the laboratory. More investigation is necessary to determine experimentally cloud point, pour point and Cold Filter Plugging Point (CFPP) as a suitability assessment of these mixes in cold areas.

### 2.7. Engine test rig

Bio-diesel fuel blends with different percentage of CNSL-derived biodiesel were used in this study as shown in Table 4.

**Table 4:** Diesel engine specifications.

Make	Kirloskar TV 1 model
Type of Engine	Type 1 cylinder, 4 stroke Diesel
Power	power 3.5 kW at 1500 rpm
Stroke	110 mm
Bore	87.5mm
Cubic Capacity	0.661 liters
Compression Ratio	17.5:1
Max.Speed	2000 rpm
Min.idle Speed	750 rpm
Min.operating Speed	1200 rpm
Valve Clearance Inlet	0.18 mm
Valve Clearance Exhaust	0.20 mm
Lub.oil pump delivery	6.50 lit/min
Sump Capacity	2.70 liter
Connecting rod length	234 mm
Air box	M S Fabricated with orifice meter and manometer
Type of Cooling	Water Cooling
Dynamometer	Eddy Current
Dynamometer Loading Unit	Constant speed, Supply 230V AC
Manometer	Range 100-0-100 mm, Type U tube
Fuel tank	Capacity 15 lit with glass fuel metering column
Overall dimensions	W 2000 x D 2500 x H 1500 mm

Biodiesel made up 10, 20 and 30% of the volume of blends B10, B20 and B30 respectively. The trials were performed on a single cylinder water cooled direct injection diesel engine. Details of the engine are shown in Fig 8. The diesel engine was mounted on an eddy-current dynamometer and operated at a constant 1500 rpm as shown in Fig 9.

**Fig 8:**
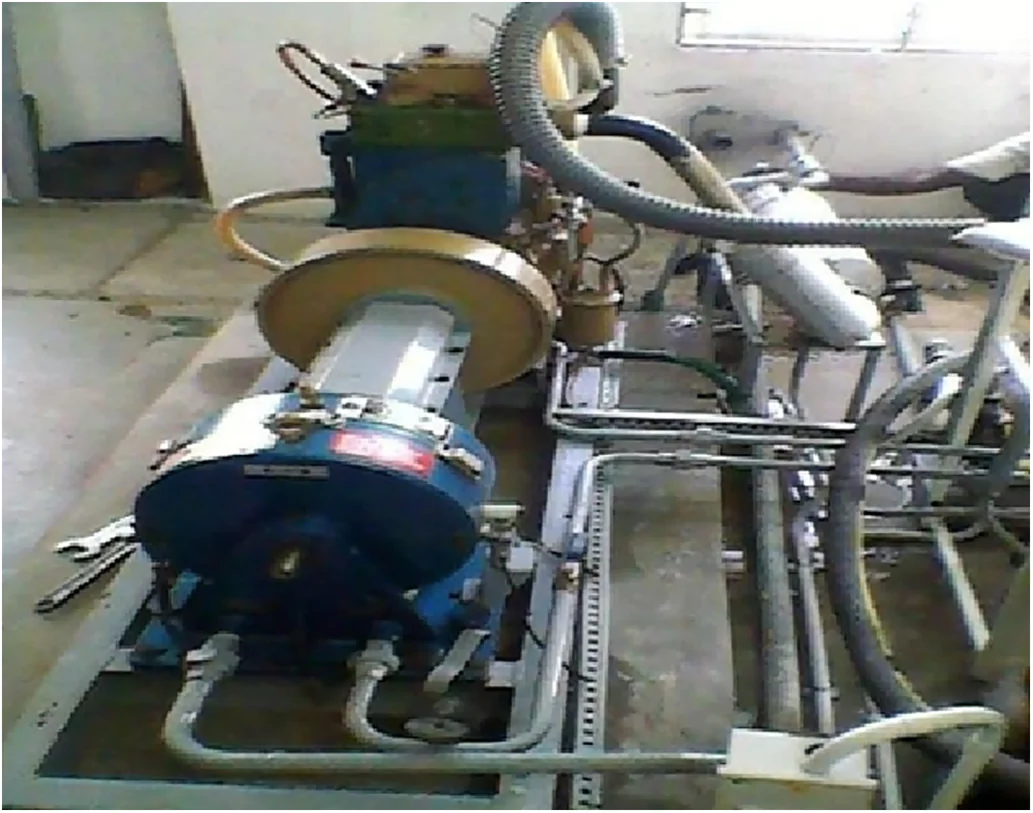
Diesel Engine with dynamometer loading unit.

**Fig 9:**
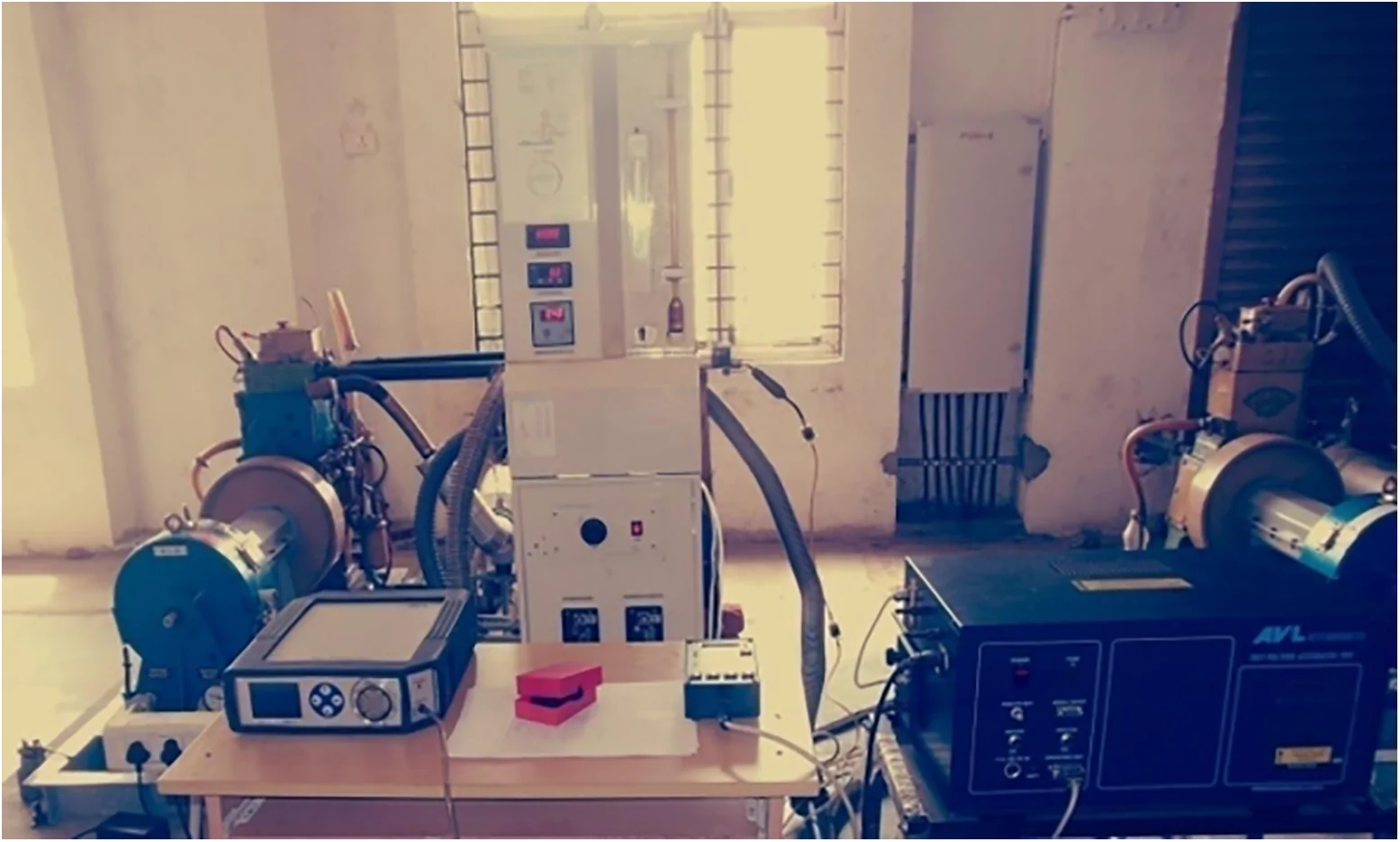
Diesel engine test setup.

#### 2.8.1. Experimental procedure.

The engine was tested in five different load scenarios (20, 40, 60, 80 and 100 kg) at constant 1500 rpm during each test. The fuel consumption was measured by the volumetric fuel consumption method by measuring the time to consume 10 cc of fuel. Braking power, brake thermal efficiency, overall fuel consumption and brake specific fuel consumption were calculated using standard engine performance formulas. Exhaust emissions such as HC, NOx and CO2 were measured with an AVL DI gas analyzer when the engine was operated at steady state conditions. All the experiments were repeated thrice and mean values were calculated.

#### 2.8.2. Repeatability and uncertainty assessment.

All fuel blends (Diesel, B10, B20 and B30) were tested under similar operating conditions. Each test was performed three times to ensure the accuracy and reproducibility of the experimental data. The brake power (BP), total fuel consumption (TFC), brake thermal efficiency (BTE), brake specific fuel consumption (BSFC), unburned hydrocarbon (UBHC), carbon dioxide (CO_2_) and nitrogen oxides (NOx) emissions were determined and shown in Table 5. These methods were used for further analysis and discussion.

**Table 5:** Uncertainty analysis of experimental measurements.

Parameter	Instrument	Accuracy	Uncertainty (%)
Load	Eddy Current Dynamometer	±0.5 kg	±0.50
Fuel Consumption	Burette and Stopwatch	±0.1 cc, ± 0.01 s	±1.20
Brake Power	Dynamometer	±0.01 kW	±1.00
Brake Thermal Efficiency	Calculated	—	±1.80
BSFC	Calculated	—	±1.50
HC Emission	AVL DI Gas Analyzer	±10 ppm	±2.00
NO_x_ Emission	AVL DI Gas Analyzer	±10 ppm	±1.50
CO_2_ Emission	AVL DI Gas Analyzer	±0.01 vol.%	±1.00

The uncertainty of the experimental measurements was quantified using the root-sum-square (RSS) method based on the accuracy of the measuring instruments used in the investigation. Table 4 summarizes uncertainty of the computed and measured parameters. The experiment showed satisfactory precision and reproducibility with an overall experimental error of ± 2.5%.

## 3. Results and discussion

The data presented in Figs 10–16 and Tables 6–9 are all averages of three different experiments performed under identical working settings. The repeatability of the obtained results was confirmed by the fluctuation between the repeated measurements being within the estimated limits of the experimental uncertainty.

**Table 6:** Performance characteristic of diesel.

Sl No	Load (Kg)	Time Taken for 10cc of fuel (Seconds)	B.P (Kw)	TFC (Kg/h)	BSFC (Kg/Kwh)	ŋ BTE (%)
1.	20	49	0.835	0.607	0.726	11
2.	40	42	1.73	0.72	0.416	20
3.	60	36	2.53	0.824	0.326	25
4.	80	29	3.40	1.014	0.298	27.4
5.	100	22	4.26	1.337	0.314	26

**Table 7:** Performance Characteristics B10.

Sl No	Load (Kg)	Time Taken for 10cc of fuel (Seconds)	B.P (Kw)	TFC (Kg/h)	BSFC (Kg/Kwh)	ŋ BTE (%)
1.	20	40	0.835	0.747	0.895	9
2.	40	1.73	0.95	0.62	0.549	40
3.	60	27	2.53	1.098	0.434	19
4.	80	22	3.40	1.014	0.298	21.5
5.	100	19	4.26	1.561	0.366	22

**Table 8:** Performance characteristics B20.

Sl No	Load (Kg)	Time Taken for 10cc of fuel (Seconds)	B.P (Kw)	TFC (Kg/h)	BSFC (Kg/Kwh)	ŋ BTE (%)
1.	20	40	0.835	0.755	0.904	9.5
2.	40	33	1.73	0.955	0.552	14.7
3.	60	27	2.53	1.108	0.438	19.6
4.	80	24	3.41	1.246	0.365	26.5
5.	100	20	4.26	1.474	0.346	30.2

**Table 9:** Performance characteristics B30.

S. No	Load (Kg)	Time Taken for 10cc of fuel (Seconds)	B.P (Kw)	TFC (Kg/h)	BSFC (Kg/Kwh)	ŋ BTE (%)
1	20	42	0.84	0.72	0.857	10
	40	34	1.73	0.93	0.538	15
2	60	27	2.53	1.2	0.474	20
3	80	22	3.4	1.36	0.400	22
4	100	20	4.26	1.52	0.357	24.7

**Fig 10:**
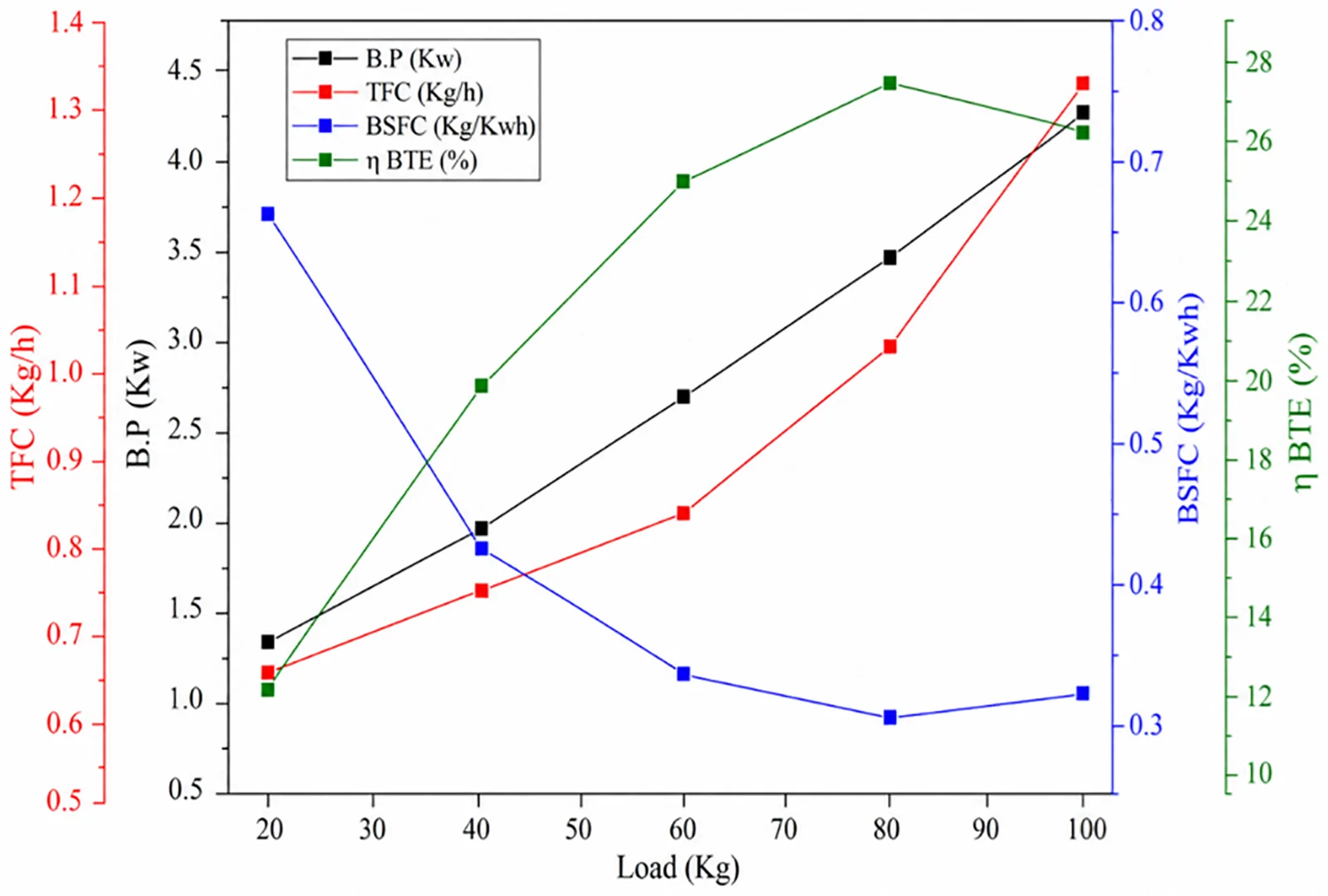
Effect of load on performance characteristics of diesel fuel.

**Fig 11:**
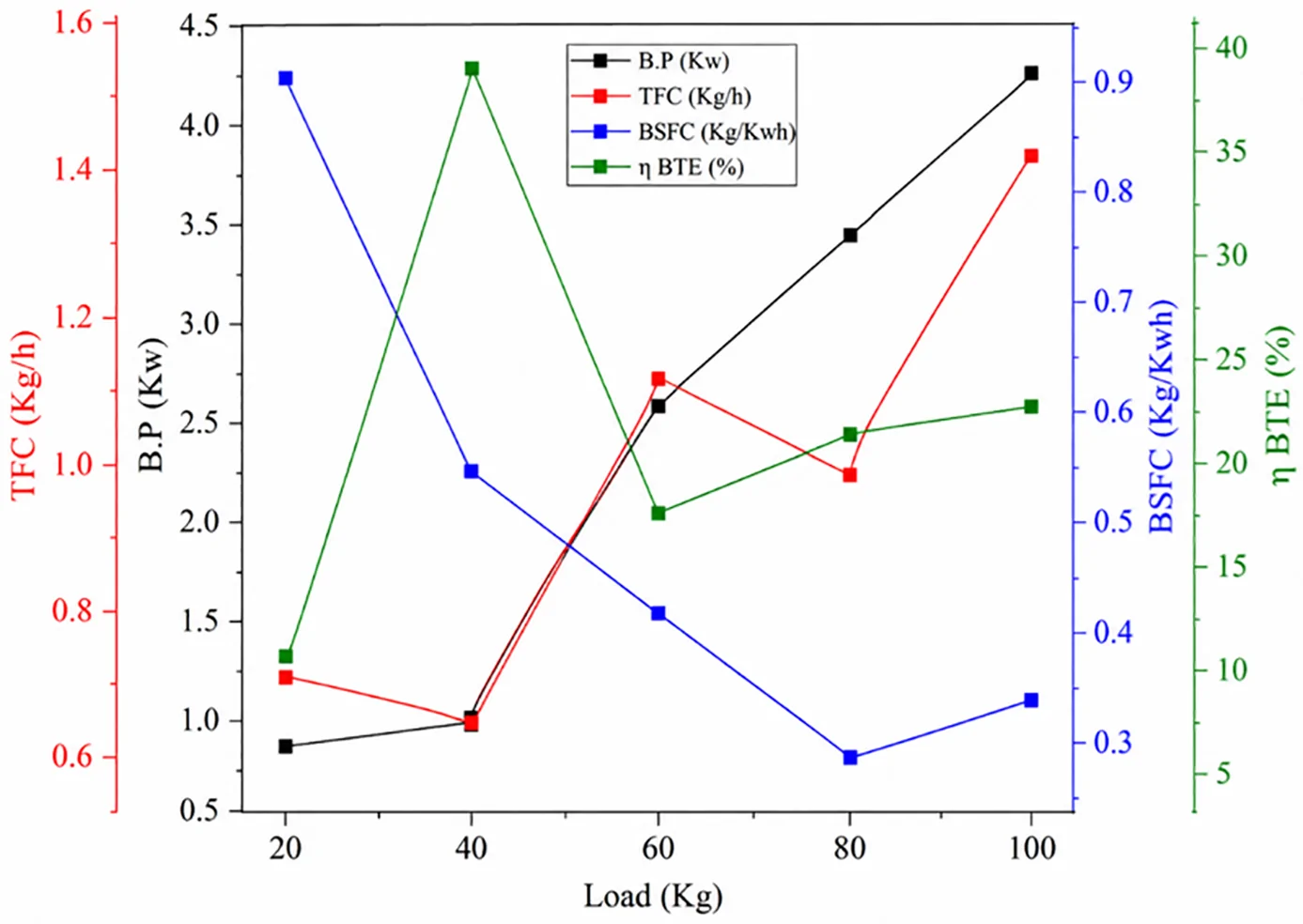
Effect of load on performance characteristics of B10 biodiesel blend fuel.

**Fig 12:**
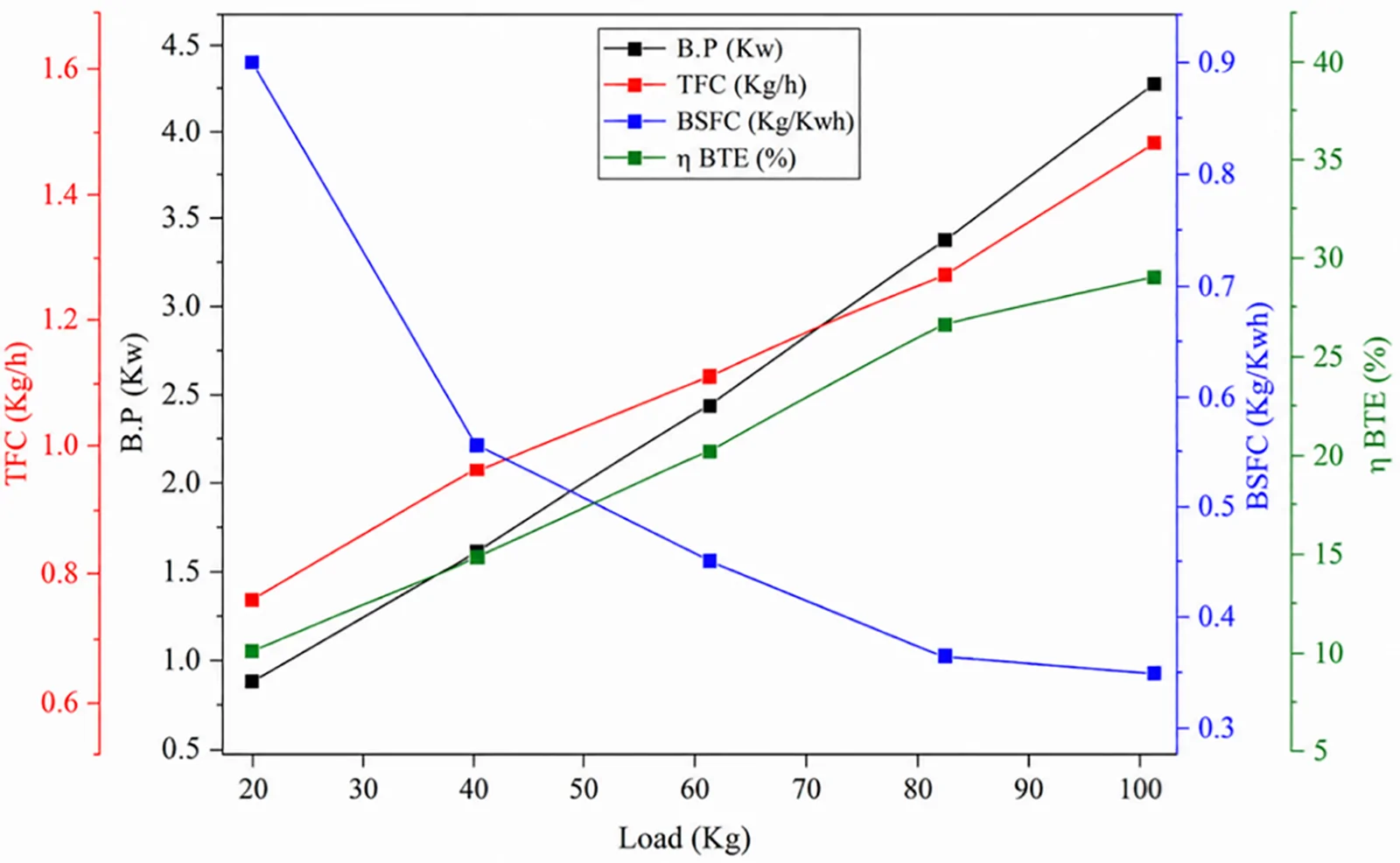
Effect of load on performance characteristics of B20 biodiesel blend fuel.

**Fig 13:**
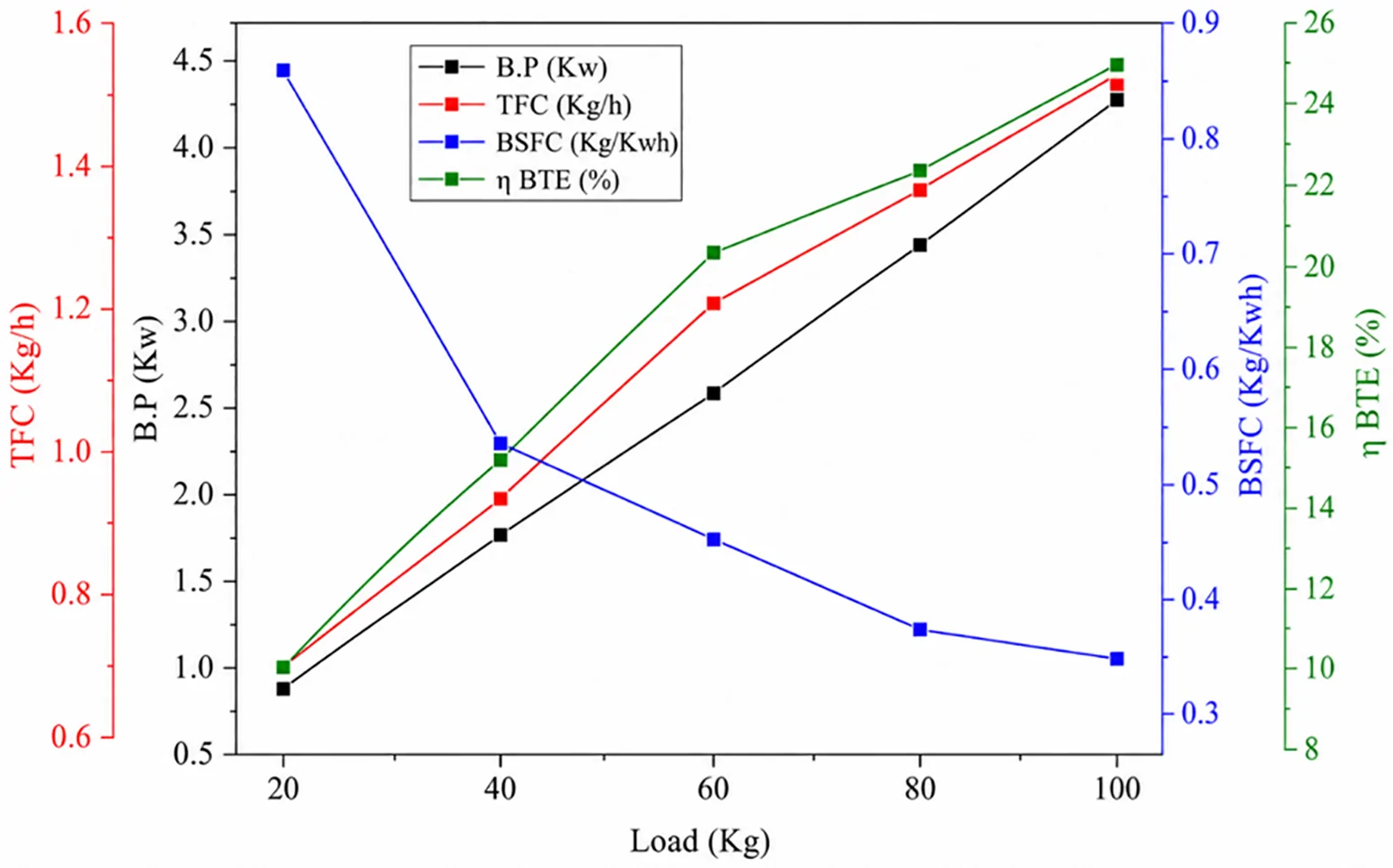
Effect of load on performance characteristics of B30 biodiesel blend fuel.

**Fig 14:**
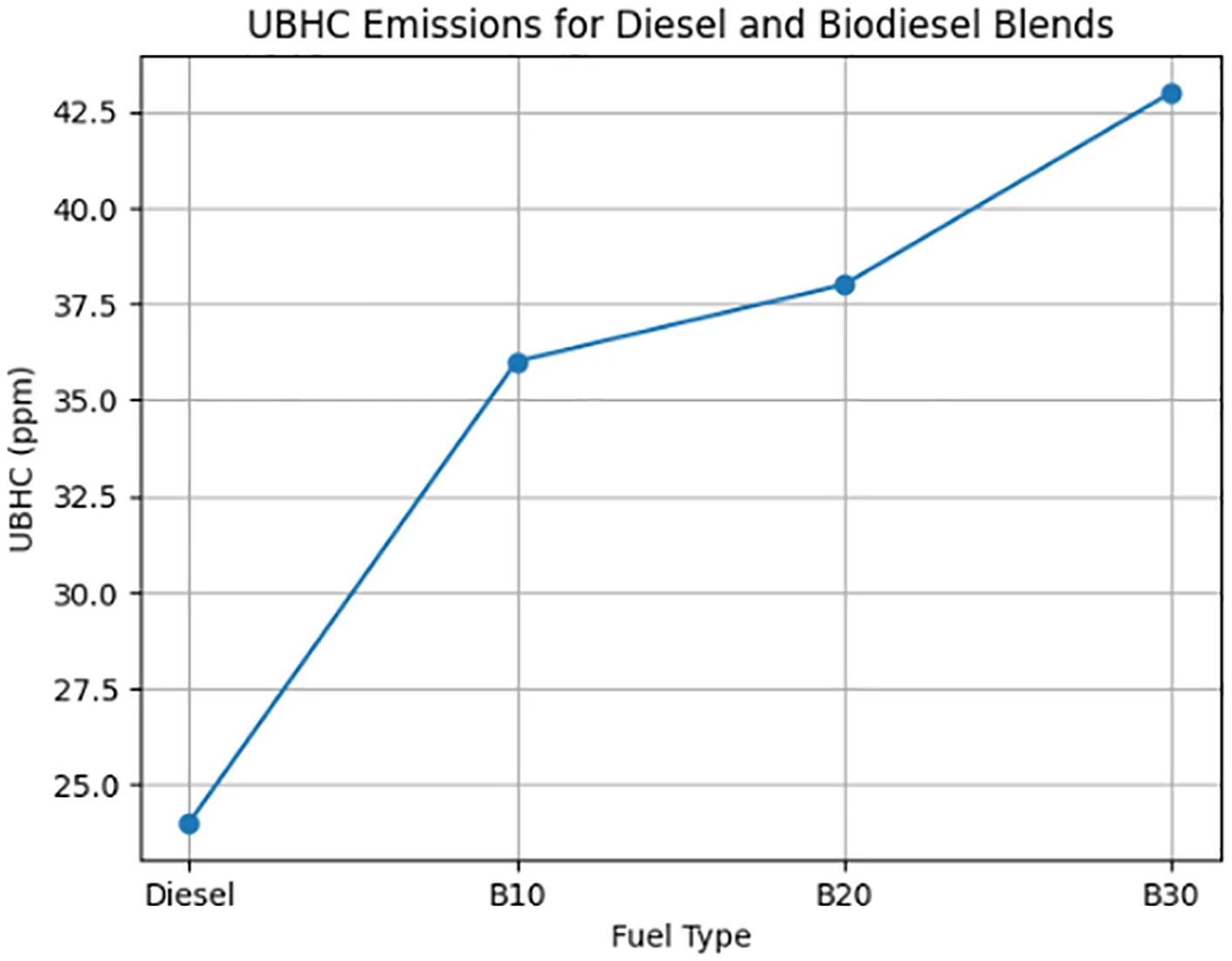
UBHC emission Vs Fuel types.

**Fig 15:**
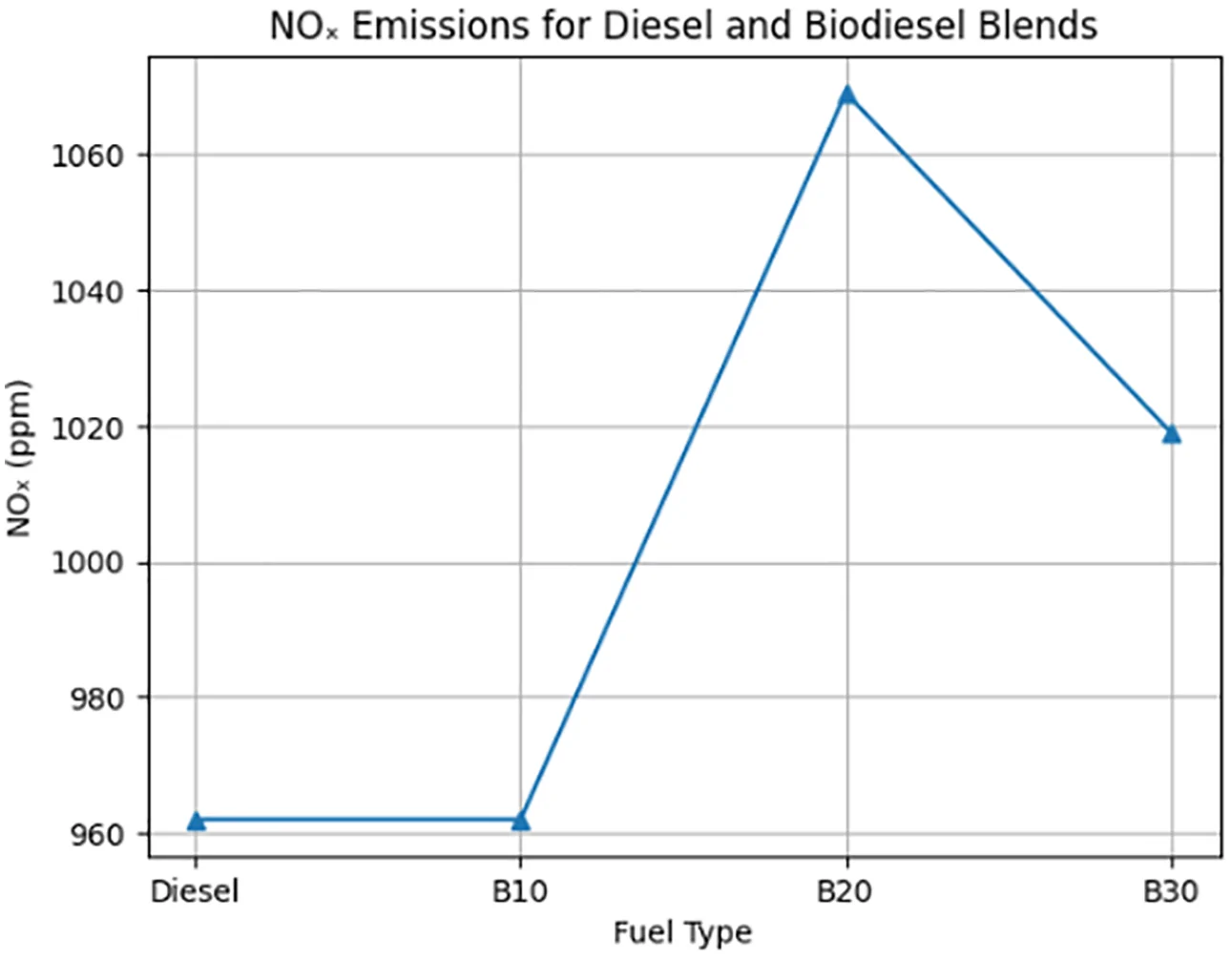
NO_x_ emission Vs Fuel types.

**Fig 16:**
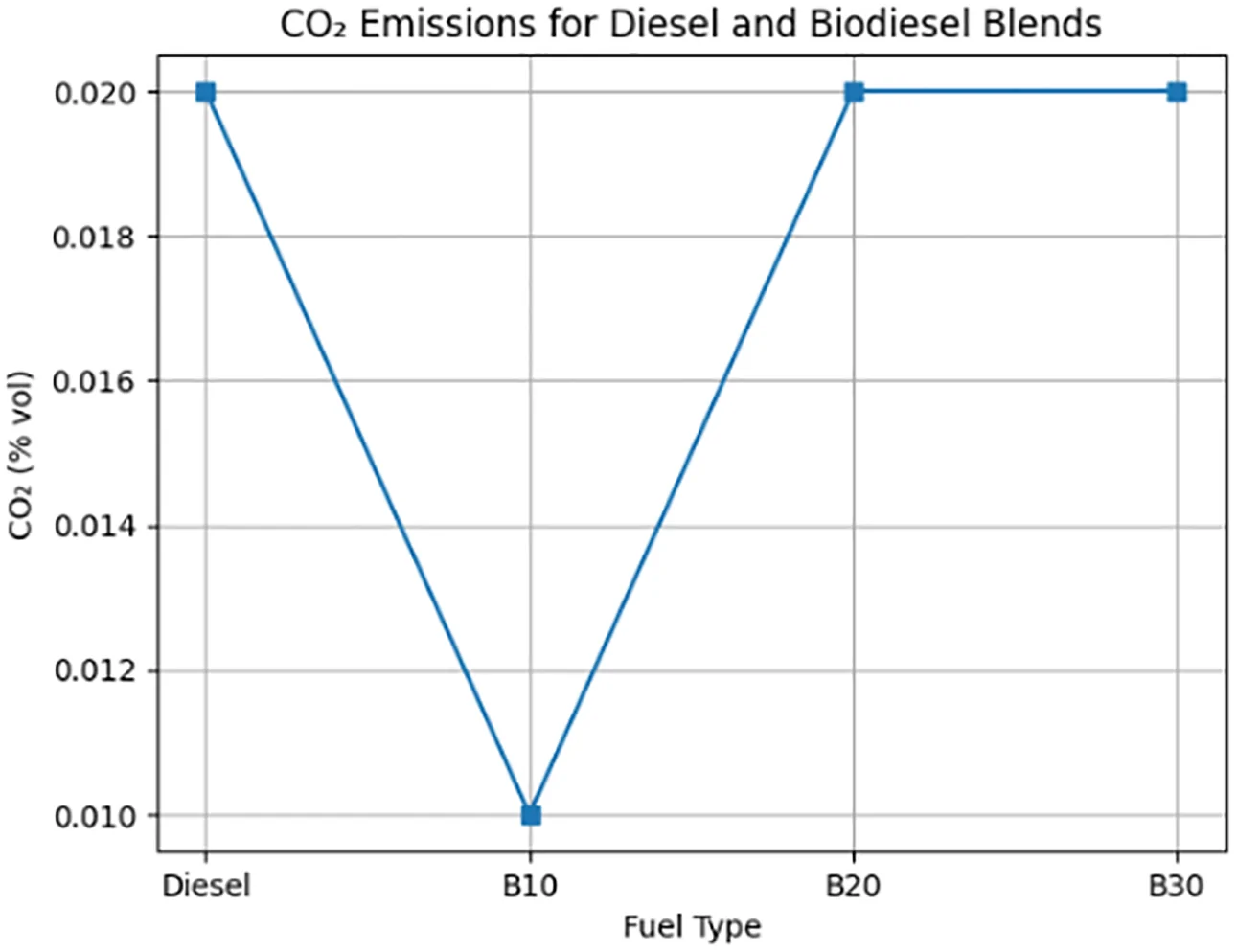
CO_2_ emission Vs Fuel types.

### 3.1. Performance characteristics of Diesel

The functioning of the diesel engine operated with pure diesel at varied load is shown in Table 5. As revealed in Table 6, the engine performance was investigated by determining the brake power (BP), total fuel consumption (TFC), brake specific fuel consumption (BSFC) and brake thermal efficiency (BTE).

#### 3.1.1. Effect of load on Brake power (BP) under diesel fuel.

As revealed in Fig 10, the braking power (BP) increases from 0.835 kW to 4.26 kW with the load increasing from 20 kg to 100 kg. The foremost cause for this increase is the higher demand on brake torque on the engine crankshaft at higher loads. As the load increases, more fuel is fed during each cycle so as to keep the engine running smoothly, therefore releasing a greater quantity of heat during combustion. Thus, the in-cylinder pressure is increased, which increases the effective pressure and the mechanical work production. The additional mechanical output is effectively used to generate braking power, as the engine speed remains constant and the friction losses are relatively constant. This causes a steady, nearly linear rise in blood pressure with enhancing the load.

#### 3.1.2. Effect of load on Total Fuel Consumption (TFC) under diesel fuel.

Fig 10 shows the total fuel consumption (TFC) that decreases from 0.747 kg/h at 20 kg load to 0.620 kg/h at 40 kg load. This reduction is owed to the enhanced combustion and utilization of the fuel supplied as the engine moves from very low-load operation to a more efficient loading condition. TFC increases slightly to 1.014 kg/h at 80 kg and then drops to 1.098 kg/h at 60 kg and again sharply rises to 1.561 kg/h at 100 kg with further increase in load. The higher fuel requirements at higher loads are associated with the higher torque demand and the proportional increase in fuel supply needed to maintain the engine at a constant speed. In general, the trend is that TFC increases with load with only minor variations due to variations in operating conditions and combustion.

#### 3.1.3 Effect of load on Brake Specific Fuel Consumption (BSFC) under diesel fuel.

As shown in the Fig 10, the BSFC is reduced from 0.895 kg/kWh at 20 kg load to 0.549 kg/kWh at 40 kg and then to 0.434 kg/kWh at 60 kg. With increasing engine load this decrease is explained by better fuel utilization and better conversion of the combustion energy produced into usable brake power. Also, the fuel efficiency is higher with the load since the frictional losses are constant in proportion and are distributed over a greater braking power output. The BSFC has the lowest value of 0.298 kg/kWh at 80 kg loading which indicates the most effective operating condition. However, BSFC increases slightly to 0.366 kg/kWh for 100 kg load. This may be because a higher fuel requirement and a lower combustion efficiency for the maximum loading condition. In general, BSFC shows a decreasing trend with increase in load up to 80 kg and then at 100 kg it increases slightly.

#### 3.1.4. Effect of load on Brake Thermal Efficiency (BTE) under diesel fuel.

As shown in Fig 10, BTE increases with engine load from 9% at 20 kg to 40% at 40 kg. This notable improvement is due to the improved consumption of the fuel energy and the reduction in the qualified contribution of the frictional and other fixed power losses at the higher loads. However, at 60 kg, BTE is 19% and then it slowly increases to 21.5% and 22% at 80 and 100 kg respectively. The variations may be due to differences in fuel-air mixing, combustion properties and heat-transfer losses at various loading conditions. In total, 40 kg load gives the best BTE of 40% and efficiency is almost constant at higher loads.

### 3.2. Performance characteristics of bio fuel blends (B10)

Table 7 represents the performance of bio fuel B10 blends (900 ml of diesel + 50 ml of rice bran oil + 25 ml of calophyllum inophyllum + 25 ml of cashew shell oil) under varying loads.

#### 3.2.1. Effect of load on Brake power (BP) under B10 biodiesel blend fuel.

The brake power (BP) gradually increased from about 0.85 kW at 20 kg load to 4.26 kW at 100 kg load, as shown in Fig 11. The increase of blood pressure is mainly due to increased brake torque demand on the engine crankshaft with increasing load. The fuel used in this work is a B10 biodiesel blend which contains 900 ml of diesel, 50 ml of rice bran oil, 25 ml of Calophyllum inophyllum oil and 25 ml of cashew shell oil. As the load enhances, excess fuel is given to each cycle to keep the engine speed up and to compensate for the increased power requirement. The blend of biodiesel provides sufficient combustible energy to support combustion, which results in higher in-cylinder pressure and effective power generation. Thus, the greater mechanical work developed in the power stroke is transformed into an increased brake power. This causes a continuous rise of the blood pressure with the rise of the load.

#### 3.2.2. Effect of load on Total Fuel Consumption (TFC) under B10 biodiesel blend fuel.

The TFC initially decreases from about 0.65 kg/h at 20 kg load to 0.60 kg/h at 40 kg load as shown in Fig 11. The reduction could be attributed to the improved utilization of the fuel and the combustion at the intermediate loading conditions. With further load TFC increases to about 1.10 kilogram/h at 60 kg and then decreases slightly to about 1.00 kg/h at 80 kg, before increasing to about 1.40 kg/h at 100 kg. The increase in the consumption at higher loads is mainly due to the increase in the torque and power demand which requires a larger amount of B10 blend to keep the engine speed up. The oxygen-containing parts of biodiesel can help with combustion, but they have less calorific value than diesel and may require an increase in the fuel supplied to meet the power demand required.

#### 3.2.3. Effect of load on Brake Specific Fuel Consumption (BSFC) under B10 biodiesel blend fuel.

From Fig 11, it can be seen that BSFC diminishes as the load enhanced from 30 to 40 kg. At 20 kg, BSFC is about 0.91 kg/kWh, at 40 kg it is 0.55 kg/kWh and at 60 kg it is 0.42 kg/kWh. At 80 kg, it drops even more to about 0.29 kg/kWh, which shows an improvement in fuel efficiency at higher engine loads. This reduction is mainly due to the increase of brake power to fuel utilization and the reduction of the effect of relatively constant frictional and auxiliary losses at higher loads. The oxygenated biodiesel components in the B10 blend may also promote further the improved fuel consumption and combustion. But at 100 kg load, BSFC is slightly increased to around 0.33 kg/kWh. This can be explained by the increased fuel requirement and possible decrease of combustion efficiency in the maximum loading conditions. Hence, the minimum BSFC is obtained at 80 kg load.

#### 3.2.4. Effect of load on Brake Thermal Efficiency (BTE) under B10 biodiesel blend fuel.

The BTE surges with the increase of the engine load. It increases from about 10% at 20 kg to 15% at 40 kg, 20% at 60 kg, 22% at 80 kg and about 24–25% at 100 kg as revealed in Fig 11. The increase in BTE with load is primarily attributed to improved conversion of the chemical energies of B10 blend into useful brake power. The relative part of the fixed power losses, such as the frictional, pumping and other losses becomes smaller in relation to the total power developed by the engine at larger loads. Moreover, the oxygenated constituents of B10 blend, rice bran oil, Calophyllum inophyllum oil and cashew shell oil, can enhance better oxidation of the fuel during combustion. Therefore, more of the supplied fuel energy is being transformed into suitable mechanical work, thus resulting in an overall increase in BTE with increasing load.

### 3.3. Performance characteristics of bio fuel blends (B20)

The B20 biofuel blend, consisting of 80% pure diesel and 20% of bio-oils (rice brain oil, Calophyllum inophyllum oil and cashew nut shell oil), was tested under various load and the outcomes are revealed in the table 8.

#### 3.3.1. Effect of load on Brake power (BP) under B20 biodiesel blend fuel.

From Fig 12, it can be detected that the BP increases gradually from about 0.86 kW at 20 kg load to 4.25 kW at 100 kg load. This is mainly because as the load increases, the brake torque demand on the engine crankshaft increases. The B20 biodiesel blend used in this study is composed of 800 milliliters of diesel, 100 milliliters of rice bran oil, 50 milliliters of Calophyllum inophyllum oil and 50 milliliters of cashew shell oil. With higher loads, more B20 fuel is required in each cycle to keep the engine speed constant and meet the higher power demand. Biodiesel’s oxygenated contents help better fuel oxidation and combustion which increases the in-cylinder pressure and produces meaningful mechanical work. when a result, when engine load increases, braking power increases linearly.

#### 3.3.2. Effect of load on Total Fuel Consumption (TFC) under B20 biodiesel blend fuel.

Fig 12 shows that the total fuel consumption (TFC) tends to increase with the engine load. The TFC increases from about 0.75 kg/h at a load of 20 kg to 0.95 kg/h at 40 kg, to approximately 1.1 kg/h at 60 kg and 1.2 kg/h at 80 kg and finally to about 1.55 kg/h at 100 kg. The increase of TFC is essentially due to the higher power and torque demand at higher loads. In such cases, more B20 mix has to be fed into the combustion chamber with each cycle to maintain the engine speed. The B20 blend may also need a larger supply of fuel to be able to achieve the required power output. B10 has a slightly lower calorific value than B20 as it has a lower percentage of biodiesel components. Therefore, the TFC increases with the engine load.

#### 3.3.3. Effect of load on Brake Specific Fuel Consumption (BSFC) under B20 biodiesel blend fuel.

The BSFC diminished significantly with enhanced load from about 0.91 kg/ kWh at 20 kg to 0.55 kg/ kWh at 40 kg and then to about 0.44 kg/ kWh at 60 kg, as shown in Fig 12. At 80 kg it falls to about 0.36 kg/kWh and at 100 kg it falls to 0.33 kg/kWh. The reduction of BSFC indicates that more of the B20 gasoline supplied is being used to produce higher engine loads. At lower load a higher proportion of the generated power is lost in frictional, pumping and other relatively constant losses, so that the fuel energy is used less efficiently. The fuel utilization per unit of brake power decreases with the rise of the load since the brake power increases significantly and the fuel consumption increases more slowly. The oxygenated components of biodiesel may also help in improved fuel utilization and combustion. Thus, the minimal BSFC is at the load of 100 kg which shows an enhancement in the fuel efficiency at the maximum load under consideration.

The BSFC of biodiesel blends is normally larger than that of standard diesel because biodiesel is denser and has a lower calorific value. This means you need more fuel for the same amount of brake power. However, the oxygen content of the biodiesel leads to more complete oxidation of the fuel thereby increasing the combustion efficiency. In the present investigation, the BSFC values of B20 blend at higher loads were nearer to diesel indicating better fuel consumption characteristics. Recent trials with Sterculia foetida biodiesel have indicated that oxygenated fuel blends can enhance combustion efficiency and reduce fuel consumption during high load running.

#### 3.3.4. Effect of load on Brake Thermal Efficiency (BTE) under B20 biodiesel blend fuel.

From Fig 12 it is seen that the BTE enhanced throughout as the engine load increases from 20 kg to 40 kg. At 20 kg load, BTE is about 10%, 15% at 40, 20% at 60 kg, about 22% at 80 kg and 24–25% at 100 kg. The increase in BTE is mainly owed to the higher brake power developed in comparison to the fuel energy input. At higher loads a larger fraction of the produced chemical energies from the B20 blend can be converted into useful mechanical work, as the fixed losses from friction and pumping are of less significance. In addition, the B20 blend also contains oxygenated components like cashew shell oil, rice bran oil and Calophyllum inophyllum oil that can potentially promote more efficient oxidation during combustion. Therefore, the BTE shows the general increasing trend with the load and achieves the maximum thermal efficiency at the 100 kg load.

In addition, the B20 blend provides the best mixture of viscosity and fuel oxygen content. The upsurge in biodiesel percentages increases the viscosity and this may have an adverse effect on the atomization characteristics. However, B20 blend retains good spray properties with the advantage of higher oxygen availability. Therefore, the development in BTE is due to the improvement in combustion quality and heat release characteristics.

#### 3.3.5. Comparison of B20 blend BTE with other non-edible biodiesel blends.

The experimental results were further validated by comparing the braking thermal efficiency with recent studies on ternary and non-edible biodiesel blends. Among all the fuels tested, B20 blend gave the best brake thermal efficiency. Therefore, a moderate biodiesel blending percentage provides the best compromise between fuel oxygen content, calorific value and spray characteristics. Similar results have been reported for blends of pine oil, hemp oil and methyl ester [30] in which intermediate mix percentages showed better combustion efficiency due to the extra oxygen in biodiesel that allowed the more complete oxidation of the fuel while maintaining acceptable atomization properties. Higher brake thermal efficiency with B20 blends has been consistently observed with several non-edible biodiesel feedstocks such as Calophyllum inophyllum, Jatropha curcas, Pongamia pinnata and Sterculia fetida compared to higher biodiesel percentages. Contrary to the above, increase in the percentage of biodiesel more than the optimum percentage generally results in reduction of the brake thermal efficiency due to the increased viscosity, decreased volatility, increased fuel consumption and decreased heating value, all of which adversely affect the fuel atomization and combustion.

The present results are in good agreement with these well-established combustion characteristics, and the ternary rice bran oil–Calophyllum inophyllum–CNSL biodiesel blend at B20 concentration is an optimum balance between increased oxygen availability and acceptable fuel properties, resulting in enhanced combustion efficiency without any engine modifications.

### 3.4. Performance characteristics of B30 biofuel blend

The performance characteristics of 70% diesel and 30% of biofuel (rice bran oil, calophyllum inophyllum oil and cashew nut shell oil) in the diesel engine is shown in the Table 9.

#### 3.4.1. Effect of load on Brake power (BP) under B30 biodiesel blend fuel.

From Fig 13 that the brake power (BP) increases gradually from about 0.86 kW at 20 kg load to 4.25 kW at 100 kg load. In this investigation, the B30 biodiesel mixture was used as the fuel source that contained 700 ml of diesel, 150 ml of rice bran oil, 75 ml of Calophyllum inophyllum oil, and 75 ml of cashew shell oil. The increase in brake torque demand on the engine crankshaft is the foremost reason for the load dependency of BP. With increasing load more B30 fuel is provided each cycle to maintain engine speed and meet the increased power demand. The oxygenated nature of the components of the biodiesel allows for efficient fuel oxidation and combustion which leads to higher in-cylinder pressure and more useful mechanical work. Thus with increase of the load the braking power is increasing steadily.

#### 3.4.2. Effect of load on Total Fuel Consumption (TFC) under B30 biodiesel blend fuel.

Normally, as engine load increases, total fuel consumption (TFC) increases as shown in Fig 13. TFC is approximately 0.72 kg/h at 20 kg, increasing to approximately 0.95 kg/h at 40 kg, 1.10 kg/h at 60 kg, 1.20 kg/h at 80 kg and approximately 1.55 kg/h at 100 kg. The higher power and torque needed at higher loads causes the fuel consumption to increase. To maintain the engine at a constant speed, more B30 mix is needed to power each combustion cycle. The quantity of fuel needed to produce the required power output may be much higher. B30 has a lesser calorific value than normal diesel owed to the higher proportion of biodiesel derived components. Thus, TFC increases as load increases.

#### 3.4.3. Effect of load on Brake Specific Fuel Consumption (BSFC) under B30 biodiesel blend fuel.

Fig 13 shows a significant reduction in the BSFC with the intensification in the load on the engine. It is 0.91 kg/kWh at 20 kg load and it decreases to 0.54 kg/kWh at 40 kg and then it is about 0.44 kg/kWh at 60 kg, 0.37 kg/kWh at 80 kg and 0.33 kg/kWh at 100 kg. This reduction demonstrates that the B30 blend is being used more efficiently as the engine is loaded. The BSFC is higher at low load because the fixed losses of pumping, frictional and others are a higher proportion of the total power generated. With the increase of load the fuel utilization per unit of brake power decreases. This is because the brake power increases rapidly while the fuel consumption increases comparatively slowly. The B30 blend oxygenates may also improve fuel utilization and combustion. Thus minimum BSFC is obtained at 100 kg load.

#### 3.4.4. Effect of load on Brake thermal efficiency (BTE) under B30 biodiesel blend fuel.

Brake thermal efficiency (BTE) typically rises with engine load, from about 10% at 20 kg to 15% at 40 kg, 20% at 60 kg, 22% at 80 kg, and 25% at 100 kg as shown in Fig 13. The enhancement in BTE is attributed mostly to the B30 blend which has been found to convert the chemical energies into the usable brake power more efficiently at higher loads. At low load a large part of the fuel energy is consumed in frictional, pumping and other losses which are relatively constant. These fixed losses decrease with the increase of power output of the load and improve the thermal utilization as the load increases. Moreover, the oxygenated components of B30 blend such as rice bran oil, cashew shell oil and Calophyllum inophyllum oil can increase the accessibility of oxygen during combustion. Therefore, BTE increases with load and it reaches maximum of around 25% at load of 100 Kg.

### 3.5. Emission performance of bio-diesel blends

The emission performance of bio-diesel blends (B10, B20 and B30) were analysed in terms of unburned hydrocarbons (UBHC), oxides of nitrogen (NO_x_) and carbon dioxide (CO_2_). The declared emission levels of UBHC, NO_x_ and CO_2_ for each fuel blend are the average values for all tested load situations (20–100 kg).

#### 3.5.1. Unburned Hydrocarbon (UBHC).

The emission of UBHC is gradually increased with increasing the bio-oil content as associated to pure diesel as exposed in Fig 14. The UBHC of B30 biodiesel blend is 44.18% greater while compared to conventional pure diesel. This was owed greater blend biodiesel affects evaporation and fuel spray atomization owed to its higher viscosity and reduced volatility of biodiesel components. This leads to inefficient combustion and an incorrect air-fuel combination in biodiesel mixes, which raises the UBHC. Furthermore, the extended ignition delay of biodiesel blends is the cause of the increase in UBHC.

#### 3.5.2. Oxides of Nitrogen (NO_x_) Emission.

The NO_x_ emission of the biodiesel blends is shown in Fig 15. Form the Fig 15, it seems that as among all the biodiesel blends, the B10 biodiesel blend shows highest NO_x_ reduction as associated to pure diesel. This is owed to the diminished premixed heat discharge and average ignition temperature of B10 biodiesel blend. On the other hand, increasing the biodiesel portion beyond B10 level, the oxygen availability increases which enhances the combustion duration of flame temperature, resulting excess NO_x_. The oxygen enrichment and heat absorption during fuel vaporization is shown by the non-linear change for B30 biodiesel blend.

The dwelling time of combustion gases in the high temperature reaction zone, the oxygen content and the combustion temperature all exert a considerable influence on the formation of nitrogen oxide. Biodiesel fuels have oxygen in their molecular structure which results in more complete combustion and higher local flame temperatures. Therefore, depending on the blend ratio, combustion temperature, oxygen availability, and residence time, biodiesel blends may produce higher or lower NOx emissions than conventional diesel.

In the present investigation, NOx emissions of B10 blend were significantly lower than diesel. NOx emissions from B30 were lower than diesel but higher than B10. NOx emissions from B20 were higher than diesel. The non-monotonic fluctuation in the NOx emissions is attributed to the combined effect of the biodiesel oxygen concentration, combustion temperature and residence time of the combustion gasses in the high temperature reaction zone.

The increase in the NOx emissions for the biodiesel mixes is due to the heat NOx generation mechanism and not only the oxygenated nature of the fuel. The extended Zeldovich mechanism predicts that significant formation of NOx occurs when local combustion temperatures are above ~1800 K. The additional oxygen in biodiesel facilitates a more complete combustion, resulting in a rise in the peak flame temperature and the dwelling time of the combustion gases at high temperature. In these conditions, the oxygen radicals and nitrogen molecules are turned into nitric oxide, which is then oxidized to nitrogen dioxide. So the increase of combustion temperature and thermal NOx kinetics are the major reasons for the increase of NOx emissions. Similar results have been observed for second generation biodiesel fuels emulsified with hydrogen enriched nanoparticles, where improved combustion efficiency increased thermal NOx creation despite reductions in other exhaust emissions [31].

#### 3.5.3. Carbon dioxide (CO_2_) Emission.

The emission of CO_2_ shows variation across the different portion of biodiesel blends, B10 biodiesel blend shows 0.01% vol, B20 and B30 biodiesel blend shows 0.02% vol. However, the pure diesel shows 0.02% vol which is shown in Fig 16. However, the B10 blend produced 50% lower CO_2_ emission than the pure diesel. This is due to that the B10 blends utilizes excess carbon content at enhanced combustion of biodiesel. However, at higher biodiesel blend ratios, the oxidation of carbon increased which increases the CO_2_ emission. Nevertheless, the CO_2_ emission was limited by increasing the bio-oil ratio sue to the presence of oxygen content within its molecular structure. CO_2_ emissions were observed to occur during combustion of biodiesel, but the emitted carbon is derived from biomass feedstocks which previously absorbed atmospheric CO_2_ during their growth. Biodiesel-derived CO_2_ is therefore considered biogenic carbon and contributes less to net greenhouse gas accumulation than fossil diesel. This results in lower lifecycle carbon emissions of biodiesel blends even though tailpipe CO_2_ measurements are similar. CO_2_ emission trends indicate a more complete oxidation of carbon containing compounds in biodiesel combustion. The carbon in the biodiesel is derived from sustainable biomass feedstocks which absorb carbon dioxide from the atmosphere during growth, even though the exhaust gases may contain somewhat elevated concentrations of CO_2_. The CO_2_ emitted during the combustion of biodiesel is generally considered to be biogenic carbon and contributes less to the net accumulation of greenhouse gases than diesel fuel derived from fossil fuels.

Similar observations have been reported in recent biodiesel studies with Calophyllum inophyllum biodiesel and Sterculia foetida oil, where an increase in oxidation led to a marginally higher CO_2_ emissions along with a decrease in the carbon footprint of the fuel on a life-cycle basis.

### 3.6. Comparison of performance characteristics of diesel, B10, B20 and B30 biodiesel blends

All the fuels including pure diesel and the biodiesel blends exhibits similar BP through the entire load range. The pure diesel produces higher BP sue to it higher volatility and calorific value. The biodiesel blends B10 and B20 shows BP values very close to diesel, however B30 blend shows slightly reduced BP, owed to lower heating range and enhanced viscosity. This shows that the biodiesel blends have negligible effect on BP.

According to TFC value, the diesel shows lowest value owed to its higher density. However, the biodiesel blends show higher TFC as the blend ratio augmented. This is owed to the minimal calorific value of all biodiesel blends which utilize excess amount of fuel for similar power output. On the other hand, BSFC decreased with enhancing the load for all the fuels. The diesel alone shows the lowest BSFC value sue to its high combustion rate. The B20 blend alone shows closer BSFC value to diesel fuel at higher load. This is owed to enhanced combustion which is promoted by oxygenated nature of biodiesel blend. According to BTE value, as the load increases the BTE also increased. The diesel fuel shows high efficiency across all loads when compared to all the biodiesel blends. However, the B20 blend shows higher BTE value at 100 kg load as related to diesel fuel. This is owed to the oxygenated nature of B20 blend where the atomization is increased and resulting more heat utilization at higher loads. Overall, the B20 biodiesel blend is suggested as best performance as competed with other biodiesel blends. Because, the B20 biodiesel blend shows similar BP, adequate BSFC performance at higher load due to its improved combustion, higher BTE value at higher load.

Generally, the cashew nut shell liquid (CNSL) biodiesel possesses better lubricity than the fossil diesel, which results in less wear of the fuel injection system. However, prolonged engine operation with high biodiesel percentages may result in injector deposits, nozzle coking and carbon deposition on the surfaces of the combustion chamber owing to the presence of oxygenated chemicals and comparatively higher viscosity. Compatibility with the fuel system is also influenced by the oxidative stability of biodiesel, which can affect rubber seals and elastomeric components during long-term storage or continuous engine operation. In the present study, no operational abnormalities like injector clogging, unstable combustion or fuel filter blockage have been observed in the engine, as the engine was run with B10, B20 and B30 blends under controlled laboratory conditions only. Therefore, long-term durability studies are recommended before large scale commercialization although the evaluated blends showed satisfactory short-term compatibility with the traditional diesel engine.

## 4. Conclusions

This study evaluated engine function and emissions on a single-cylinder, water-cooled, four-stroke direct-injection diesel engine using diesel fuel modified with different biodiesel blend

- Maximum BTE values at the highest engine load are 22.0% for B10, 30.2% for B20 and 24.7% for B30 when compared to 26.0% for neat diesel. Among the tested biodiesel blends, the blend B20 exhibited the highest BTE of 30.2%, which is about 16.2% higher than that of neat diesel.
- For the mixed biodiesel blends, the measured BSFC values were 0.36 kg/kW·h (B10), 0.34 kg/kW·h (B20), and 0.35 kg/kW·h (B30), whereas conventional diesel recorded 0.31 kg/kW·h.
- Among the assessed biodiesel blends, B20 showed the best overall performance, particularly at higher engine loads, due to its relatively good balance between fuel oxygen content, calorific value, and combustion properties.
- UBHC emissions enhanced with the biodiesel content in the blends, registering 36 ppm (B10), 38 ppm (B20), and 43 ppm (B30) relative to diesel fuel.
- NO_x_ emissions for the blends were measured at 962 ppm (B10), 1069 ppm (B20), and 1019 ppm (B30), while neat diesel produced 1092 ppm. The B10 blend exhibited the largest decrease, with NOx levels reduced by roughly 11.9% relative to diesel.
