# Supplementary material for: Performance and emission analysis of sustainable rice bran-Calophyllum-CNSL biodiesel blends in a conventional diesel engine
Source: PLoS One. 2026 Oct 30;21(10):e0360637. doi: 10.1371/journal.pone.0360637 (PMC13722191; doi:10.1371/journal.pone.0360637)
Supplement: Supplementary file 1 [file pone.0360637.s002.docx]

**Manuscript Title : Combustion Performance and Emission Analysis of Sustainable Rice Bran-Calophyllum-CNSL Biodiesel Blends in a Conventional Diesel Engine**

**Paper ID	: PONE-D-26-08657**

**Response to Reviewers Comments**

Submitted to the Editorial Office

Dear Sir,

The authors of this manuscript (**PONE-D-26-08657**) would like to thank the editor for his constructive suggestion for the improvement of this manuscript. Also, the authors like to thank the anonymous reviewers for spending their time and effort to review the manuscript and offering their valuable comments, which helped us to improve the manuscript. All the modifications suggested by the reviewer, in either explanation or answer is prepared and incorporated in the revised manuscript. Reviewer comments are highlighted in yellow colour in the updated manuscript.

**Reviewer comments:**

**Comments for the Author:**

Reviewer #1:

1. Authors should include uncertainty analysis and repeatability.

The authors appreciate the insightful suggestion of the reviewer. The values given represent the average values of the measurements taken in the three repetitions of the experimental tests under the same operating conditions. The updated publication also includes an uncertainty analysis based on measurement errors and instrument specifications.

2. Octane number is listed for diesel engine fuels; however, octane number is relevant to SI engines, whereas cetane number is appropriate for CI engines.

The authors are grateful to the reviewer for pointing out this issue. The octane number is not a good fuel quality indicator for compression ignition engines, but it is relevant for spark ignition engines. The discussion has been revised accordingly to refer to cetane number, viscosity, density and calorific value and the octane number data have been deleted from Table 2.

3. Reported viscosity values appear unusually low.

The authors thank the reviewer for noticing this observation. There is a typographical error in the quoted viscosity value of 0.928 mm^2^/s for the oil of Calophyllum inophyllum. The value has been corrected to measured and published data.

4. No calorific value data are provided; for discussing BSFC and BTE, it will help.

The authors agree with the reviewer. Now, due to the significant impact of the fuel energy content on brake thermal efficiency and brake-specific fuel consumption, the calorific value data is added to the updated document.

5. Results focus on performance parameters. Combustion analysis should be included.

The authors thank the reviewer for this comment. Direct combustion parameters such as cylinder pressure or heat release rate were not measured in the present study. Thus, the discussion has moved away from direct combustion analysis to combustion behavior that can be inferred from engine performance and emission characteristics.

6. CO₂ from biodiesel is biogenic carbon and should be discussed within a carbon-cycle context.

The authors thank the reviewer for the valuable feedback. A discussion on carbon neutrality of CO_2_ emissions from biodiesel has been included into the revised manuscript (in the section 4.5.3).

7. Whether emissions were averaged over all loads.

The authors thank the reviewer for the request of clarification. The emission levels shown in Figures 14-16 are the average of measurements taken over all engine load conditions tested. This has now been clarified in the revised MS.

8. The title includes “Combustion Performance,” yet no combustion is presented.

Authors agree with the reviewer. Since experimental measurements of direct combustion parameters like cylinder pressure, ignition delay, heat release rate or combustion duration were not performed, the title has been modified to reflect the actual scope of the work.

Revised title: Performance and Emission Analysis of Sustainable Rice Bran-Calophyllum-CNSL Biodiesel Blends in a Conventional Diesel Engine

9. Although diesel is used as the reference fuel, its specification is not provided.

The authors thank the reviewer for his comment. In the revised manuscript, Table 3 provides a detailed description of the reference diesel fuel used in the studies. In addition, Section 2.6.4 (Blend Preparation) includes details on the origin of the diesel fuel and its compliance with standard diesel fuel specifications.

Reviewer #2: The manuscript demonstrates significant relevance to both the scientific and industrial communities and holds potential archival quality; however, minor revisions are recommended to improve its overall clarity and impact.

1. The introduction effectively sets the stage but should be more focused on clearly articulating the research objectives and hypotheses. The literature review, while comprehensive, would benefit from the inclusion of more recent studies, particularly "Evaluating the performance, combustion, and emission characteristics of decanol-enhanced sterculia foetida biodiesel in diesel engines. Results in Engineering, 26, 104936.”

The authors thank the reviewer for the insightful recommendation. The revised Introduction section clarifies the research objectives and working hypothesis. The new language clearly specifies the predicted effects of ternary biodiesel blends on engine performance and emission characteristics. Also, as per the reviewer suggestion, the literature has been incorporated in the revised manuscript.

2. The methodology is well-structured, but it lacks detailed descriptions of data collection and analysis procedures, which are essential for replicability. Additionally, the rationale for the chosen sample size is insufficiently justified; incorporating a statistical power analysis or referencing prior research would strengthen this aspect, use 'Performance and emission analysis of sterculia foetida biodiesel enhanced with butanol: Combustion efficiency and emission mitigation. Results in Engineering, 25, 104586.".

The authors are grateful for the reviewer's comment. In the current version of the publication, we provide more details about the experimental measures, data gathering methods, repeatability and data processing to increase transparency and reproducibility. Also, as per the reviewer suggestion, the literature has been incorporated in the revised manuscript.

3. The explanation of statistical methods also requires more clarity to be accessible to a broader readership. Furthermore, the results and discussion section needs to elaborate on key scientific issues more thoroughly and integrate recent literature to provide stronger contextual grounding. In particular, references such as “Utilization of sterculia foetida oil as a sustainable feedstock for biodiesel production: optimization, performance, and emission analysis. Results in Engineering, 24, 103196 and Evaluating ignition improvers on performance and emissions of Calophyllum inophyllum biodiesel in turbocharged diesel engines. Results in Engineering, 24, 103664.” are recommended to enrich the discussion and align the study with current research trends.

The authors appreciate the insightful suggestion from the reviewer. The Results and Discussion section is completely revised to provide a more detailed scientific analysis of the reported engine performance and emission characteristics. Further discussion has been made on the effect of biodiesel oxygen content, fuel atomization, calorific value, combustion temperature and fuel-air mixing on the BSFC, BTE, NOx and CO2 emissions.

Furthermore, the recent literature suggested by the reviewer has been included in the discussion section to better provide context and a comparison with current research on biodiesel engines. The scientific relevance and the currentness of the manuscript have been improved by comparing the results of the present study with the results of recent studies with biodiesel fuels of Calophyllum inophyllum and Sterculia foetida.
