# Supplementary material for: Performance and emission analysis of sustainable rice bran-Calophyllum-CNSL biodiesel blends in a conventional diesel engine
Source: PLoS One. 2026 Oct 30;21(10):e0360637. doi: 10.1371/journal.pone.0360637 (PMC13722191; doi:10.1371/journal.pone.0360637)
Supplement: Supplementary file 2 [file pone.0360637.s003.docx]

**Manuscript Title : Performance and Emission Analysis of Sustainable Rice Bran-Calophyllum-CNSL Biodiesel Blends in a Conventional Diesel Engine**

**Paper ID	: PONE-D-26-08657R1**

**Response to Reviewers Comments**

Submitted to the Editorial Office

Dear Sir,

The authors of this manuscript (**PONE-D-26-08657R1**) would like to thank the editor for his constructive suggestion for the improvement of this manuscript. Also, the authors like to thank the anonymous reviewers for spending their time and effort to review the manuscript and offering their valuable comments, which helped us to improve the manuscript. All the modifications suggested by the reviewer, in either explanation or answer is prepared and incorporated in the revised manuscript. Reviewer comments are highlighted in yellow colour in the updated manuscript.

**Reviewer comments:**

**Comments for the Author:**

Reviewer #1:

1. Discuss deposit formation or fuel system compatibility associated with CNSL-containing biodiesel blends.

We thank the reviewer for this insightful suggestion. The Discussion section is enriched with a new discussion on the compatibility of CNSL containing biodiesel blends and fuel systems and deposit formation. The paper states that biodiesel is often more lubricating than diesel fuel and can reduce wear on fuel pumps and injectors.

The response has been incorporated in the revised manuscript in the section 3.6.

2. Authors should compare their BTE results with recent studies on ternary and non-edible biodiesel blends operating in conventional diesel engines LIKE "Optimization of Combustion, Performance, and Emission Characteristics of a Reactivity Controlled Compression Ignition Engine Fueled with Hemp Oil Methyl Ester and Pine Oil Blends using Response Surface Methodology".

We thank the reviewer for the thoughtful suggestion. The proposed paper is referenced not for optimization, but to compare the trends of brake thermal efficiency (BTE) with the current biodiesel studies. Moreover, as per the reviewer suggestion, the literature has been incorporated in the revised manuscript.

The updated manuscript now emphasizes the similarities in thermal efficiency and combustion behavior by comparing the observed BTE trends with recent studies on ternary and non-edible biodiesel blends.

The response has been incorporated in the revised manuscript in the section 3.2.5.

3. The effect of biodiesel oxygen content on NOx formation requires a more discussion on thermal NOx rather than a description. "Comparative analysis of a diesel engine fueled with hydrogen-enriched nanoparticle-emulsified second-generation biodiesel"

We thank the reviewer for the thoughtful comment. The discussion on NOx has been considerably updated with the inclusion of the thermal NOx formation mechanism based on the extended Zeldovich theory. In the revised manuscript, it is now clear that NOx is mainly formed due to higher combustion temperatures and longer residence times at high temperatures and not only due to oxygen content of biodiesel. Also, as per the reviewer instruction, the reference has been cited in the revised manuscript.

The response has been incorporated in the revised manuscript in the section 3.5.2.

4. Discuss cold-flow properties of the prepared blends.

We respect the reviewer's insightful suggestion. The revised manuscript now contains a discussion on the cold-flow behavior of the prepared biodiesel blends. The predicted cold-flow properties from the properties of the constituent biodiesel have been described. The cloud point, pour point and cold filter plugging point were not measured experimentally.

The response has been incorporated in the revised manuscript in the section 2.6.4.
