# Supplementary material for: Performance and emission analysis of sustainable rice bran-Calophyllum-CNSL biodiesel blends in a conventional diesel engine
Source: PLoS One. 2026 Oct 30;21(10):e0360637. doi: 10.1371/journal.pone.0360637 (PMC13722191; doi:10.1371/journal.pone.0360637)
Supplement: Supplementary file 3 [file pone.0360637.s004.docx]

Review Comments to the Author

Please use the space provided to explain your answers to the questions above. You may also include additional comments for the author, including concerns about dual publication, research ethics, or publication ethics. (Please upload your review as an attachment if it exceeds 20,000 characters)

Reviewer #1: After revision, the authors have satisfactorily addressed all reviewer comments. The manuscript is now suitable for publication.

We thank the reviewer for the recommendation.

Reviewer #3: The authors have made generally reasonable revisions in response to the previous review comments, particularly by expanding the discussions on fuel-system compatibility and deposit formation, comparison of brake thermal efficiency with recent studies, thermal NOx formation mechanisms, and cold-flow characteristics. These revisions have improved the manuscript and addressed several of the concerns raised in the previous round of review. Nevertheless, several key issues related to data consistency overall structural coherence remain unresolved. These issues should be satisfactorily addressed before the manuscript can be reconsidered for publication:

1. The authors have added a new Section 3.2.5 as a comparison for B20. However, this B20 literature comparison is placed under Section 3.2 "B10 Performance Characteristics," where the actual B20 results have not yet been presented. In addition, the heading of the immediately following Section 3.3 is incorrectly given as "B10," whereas its content in fact concerns B20. Please correct the section arrangement and headings accordingly.

We thank the reviewer for pointing out this inconsistency in our organization. We have carefully reorganized sections 3.2 and 3.3 so that the explanation of each biodiesel mix is placed in the correct section. The header of Section 3.3 has been changed from the wrong one to “Performance Characteristics of B20 Biofuel Blend” and the B20 literature comparison has been relocated to the B20 subsection after presenting and discussing the B20 experimental results. The numbering of the respective sub-paragraphs has been revised to bring uniformity in the work. The heading has been updated under the section 3.3 as 3.3.5.

2. The text states that the NOx emissions of both B20 and B30 are higher than those of diesel. In Fig. 15, diesel and B10 are both approximately 962 ppm, B20 approximately 1069 ppm, and B30 approximately 1019 ppm. These values do not correspond to those reported in the Conclusions.

We thank the reviewer for the discrepancy. We have revised the discussion and conclusions in light of our re-evaluation of the NOx emissions data shown in Figure 15. The corrected average NOx emissions for the blends were measured at 962 ppm (B10), 1069 ppm (B20), and 1019 ppm (B30), while neat diesel produced 1092 ppm. So B30 is less than diesel but more than B10 and B20 is more than diesel. In this way, the mistake on the trend of NOx has been corrected.

This has bee updated under the section 3.5.2 and in the conclusion

3. The fuel blends given in the text are only B10, B20 and B30, and the engine test section explicitly states that the test fuels were diesel, B10, B20 and B30. Nevertheless, the Conclusions section presents test results corresponding to B40.

We thank the reviewer for this comment. The reference to B40 in the Conclusions was a typographical error, as B40 is not an experimentally studied fuel blend. Following the experimental approach, the inquiry was limited to neat diesel, B10, B20 and B30 mixes only. The incorrect reference to B40 has been removed from the Conclusions and the sentence has been changed accordingly so that it will keep consistent with the experimental scope of the study.

4. Please check for citation mismatches. For example, the text cites Ref. [16] in support of the "performance and emissions of waste frying oil methyl ester in a diesel engine at a higher compression ratio," whereas Ref. [16] is in fact a study on the co-pyrolysis kinetics of waste tyre and walnut shell.

We thank the reviewer for this valuable suggestion. Reference list and citations in the manuscript were reviewed. We checked Ref. [16] which is a research paper on thermogravimetric properties and co-pyrolysis kinetics of scrap tire and walnut shell and it did not support the claim of waste frying oil methyl ester and increased compression ratio. So, we have taken out the unfounded and misattributed statement. We also looked at surrounding references to confirm that the literature cited supports the claims it is used for.

The reference [16] is a study of co-pyrolysis, not a study of biodiesel engines as listed in the references. This statement is not supported by the given Ref. [16] It is left out altogether.

5. Some of the BSFC values reported in the paper are inconsistent with the TFC and BP values given in the tables. The authors should further verify the relevant calculations.

The BSFC values reported in the manuscript were carefully rechecked using the corresponding total fuel consumption (TFC) and brake power (BP) values given in the experimental tables. The corresponding figures and discussions have also been reviewed and updated to ensure full consistency between TFC, BP and BSFC throughout the revised manuscript. We would like to thank the reviewer for the help in improving the accuracy and consistency of the reported results.
