# Supplementary material for: Performance and emission analysis of sustainable rice bran-Calophyllum-CNSL biodiesel blends in a conventional diesel engine
Source: PLoS One. 2026 Oct 30;21(10):e0360637. doi: 10.1371/journal.pone.0360637 (PMC13722191; doi:10.1371/journal.pone.0360637)
Supplement: Peer review history

# Peer review: Decision Letter 0

**DOI:** 10.1371/journal.pone.0360637.r001

**Authors:** Tien Anh Tran

24 May 2026

Dear Dr. Dennison,

Thank you for submitting your manuscript to PLOS ONE. After careful consideration, we feel that it has merit but does not fully meet PLOS ONE’s publication criteria as it currently stands. Therefore, we invite you to submit a revised version of the manuscript that addresses the points raised during the review process.

==============================

There are comments of reviewers which require you to address all comments and update on the revised paper. Beside that, the authors must make clear the main contributions and its novelty of this paper. The significances of this study must be implemented in the conclusion.

==============================

Please submit your revised manuscript by Jul 08 2026 11:59PM. If you will need more time than this to complete your revisions, please reply to this message or contact the journal office at plosone@plos.org. When you're ready to submit your revision, log on to https://www.editorialmanager.com/pone/ and select the 'Submissions Needing Revision' folder to locate your manuscript file.

- A letter that responds to each point raised by the academic editor and reviewer(s). You should upload this letter as a separate file labeled 'Response to Reviewers'.
- A marked-up copy of your manuscript that highlights changes made to the original version. You should upload this as a separate file labeled 'Revised Manuscript with Track Changes'.
- An unmarked version of your revised paper without tracked changes. You should upload this as a separate file labeled 'Manuscript'.

If you would like to make changes to your financial disclosure, please include your updated statement in your cover letter. Guidelines for resubmitting your figure files are available below the reviewer comments at the end of this letter.

If applicable, we recommend that you deposit your laboratory protocols in protocols.io to enhance the reproducibility of your results. Protocols.io assigns your protocol its own identifier (DOI) so that it can be cited independently in the future. For instructions see: https://journals.plos.org/plosone/s/submission-guidelines#loc-laboratory-protocols. Additionally, PLOS ONE offers an option for publishing peer-reviewed Lab Protocol articles, which describe protocols hosted on protocols.io. Read more information on sharing protocols at https://plos.org/protocols?utm_medium=editorial-email&utm_source=authorletters&utm_campaign=protocols.

As the corresponding author, your ORCID iD is verified in the submission system and will appear in the published article. PLOS supports the use of ORCID, and we encourage all coauthors to register for an ORCID iD and use it as well. Please encourage your coauthors to verify their ORCID iD within the submission system before final acceptance, as unverified ORCID iDs will not appear in the published article. *Only*  the individual author can complete the verification step; PLOS staff *cannot*  verify ORCID iDs on behalf of authors.

We look forward to receiving your revised manuscript.

Kind regards,

Tien Anh Tran

Academic Editor

PLOS One

Journal Requirements:

When submitting your revision, we need you to address these additional requirements.

1.Please ensure that your manuscript meets PLOS ONE's style requirements, including those for file naming. The PLOS ONE style templates can be found at

https://journals.plos.org/plosone/s/file?id=wjVg/PLOSOne_formatting_sample_main_body.pdf and

https://journals.plos.org/plosone/s/file?id=ba62/PLOSOne_formatting_sample_title_authors_affiliations.pdf

2. In the online submission form, you indicated that your data is available only on request from a third party. Please note that your Data Availability Statement is currently missing the name of the third party contact or institution / contact details for the third party, such as an email address or a link to where data requests can be made. Please update your statement with the missing information.

3. Please ensure that you refer to Figure 9 in your text as, if accepted, production will need this reference to link the reader to the figure.

4. If the reviewer comments include a recommendation to cite specific previously published works, please review and evaluate these publications to determine whether they are relevant and should be cited. There is no requirement to cite these works unless the editor has indicated otherwise.

[Note: HTML markup is below. Please do not edit.]

Reviewer's Responses to Questions

**Comments to the Author**

1. Is the manuscript technically sound, and do the data support the conclusions?

Reviewer #1: Yes

Reviewer #2: Partly

**********

2. Has the statistical analysis been performed appropriately and rigorously?

Reviewer #1: Yes

Reviewer #2: Yes

**********

3. Have the authors made all data underlying the findings in their manuscript fully available?

The PLOS Data policy

Reviewer #1: Yes

Reviewer #2: Yes

**********

4. Is the manuscript presented in an intelligible fashion and written in standard English?

Reviewer #1: Yes

Reviewer #2: Yes

**********

Reviewer #1: 1. Authors should include uncertainty analysis and repeatability.

2. Octane number is listed for diesel engine fuels; however, octane number is relevant to SI engines, whereas cetane number is appropriate for CI engines.

3. Reported viscosity values appear unusually low.

4. No calorific value data are provided; for discussing BSFC and BTE, it will help.

5. Results focus on performance parameters. Combustion analysis should be included.

6. CO₂ from biodiesel is biogenic carbon and should be discussed within a carbon-cycle context.

7. Whether emissions were averaged over all loads.

8. The title includes “Combustion Performance,” yet no combustion is presented.

9. Although diesel is used as the reference fuel, its specification is not provided.

Reviewer #2: The manuscript demonstrates significant relevance to both the scientific and industrial communities and holds potential archival quality; however, minor revisions are recommended to improve its overall clarity and impact.

The introduction effectively sets the stage but should be more focused on clearly articulating the research objectives and hypotheses. The literature review, while comprehensive, would benefit from the inclusion of more recent studies, particularly "Evaluating the performance, combustion, and emission characteristics of decanol-enhanced sterculia foetida biodiesel in diesel engines. Results in Engineering, 26, 104936.”

The methodology is well-structured, but it lacks detailed descriptions of data collection and analysis procedures, which are essential for replicability. Additionally, the rationale for the chosen sample size is insufficiently justified; incorporating a statistical power analysis or referencing prior research would strengthen this aspect, use 'Performance and emission analysis of sterculia foetida biodiesel enhanced with butanol: Combustion efficiency and emission mitigation. Results in Engineering, 25, 104586.".

The explanation of statistical methods also requires more clarity to be accessible to a broader readership. Furthermore, the results and discussion section needs to elaborate on key scientific issues more thoroughly and integrate recent literature to provide stronger contextual grounding. In particular, references such as “Utilization of sterculia foetida oil as a sustainable feedstock for biodiesel production: optimization, performance, and emission analysis. Results in Engineering, 24, 103196 and Evaluating ignition improvers on performance and emissions of Calophyllum inophyllum biodiesel in turbocharged diesel engines. Results in Engineering, 24, 103664.”are recommended to enrich the discussion and align the study with current research trends.

**********

what does this mean?). If published, this will include your full peer review and any attached files.

If you choose “no”, your identity will remain anonymous but your review may still be made public.

**Do you want your identity to be public for this peer review?**  For information about this choice, including consent withdrawal, please see our Privacy Policy

Reviewer #1: No

Reviewer #2: No

**********

[NOTE: If reviewer comments were submitted as an attachment file, they will be attached to this email and accessible via the submission site. Please log into your account, locate the manuscript record, and check for the action link "View Attachments". If this link does not appear, there are no attachment files.]

To ensure your figures meet our technical requirements, please review our figure guidelines: https://journals.plos.org/plosone/s/figures

You may also use PLOS’s free figure tool, NAAS, to help you prepare publication quality figures: https://journals.plos.org/plosone/s/figures#loc-tools-for-figure-preparation.

NAAS will assess whether your figures meet our technical requirements by comparing each figure against our figure specifications.

# Author comment: Author response to Decision Letter 1

**DOI:** 10.1371/journal.pone.0360637.r002

27 Jun 2026

The detailed response sheet is enclosed herewith.

Attachment Submitted filename: Response to the reviewers.docx

# Peer review: Decision Letter 1

**DOI:** 10.1371/journal.pone.0360637.r003

**Authors:** Tien Anh Tran

15 Jul 2026

Dear Dr. Dennison,

Thank you for submitting your manuscript to PLOS ONE. After careful consideration, we feel that it has merit but does not fully meet PLOS ONE’s publication criteria as it currently stands. Therefore, we invite you to submit a revised version of the manuscript that addresses the points raised during the review process.

==============================

-->Additionally, during revision process, the authors must make significant about revised paper according to the comments below.

1. What are the novelty points of this study? The authors must indicate clearly in the introduction part.

2. The significances of influences with different fuels impacting on the diesel engine's operation and maintenance. 

==============================

Please submit your revised manuscript by Aug 29 2026 11:59PM. If you will need more time than this to complete your revisions, please reply to this message or contact the journal office at plosone@plos.org. When you're ready to submit your revision, log on to https://www.editorialmanager.com/pone/ and select the 'Submissions Needing Revision' folder to locate your manuscript file.

- A letter that responds to each point raised by the academic editor and reviewer(s). You should upload this letter as a separate file labeled 'Response to Reviewers'.
- A marked-up copy of your manuscript that highlights changes made to the original version. You should upload this as a separate file labeled 'Revised Manuscript with Track Changes'.
- An unmarked version of your revised paper without tracked changes. You should upload this as a separate file labeled 'Manuscript'.

If you would like to make changes to your financial disclosure, please include your updated statement in your cover letter. Guidelines for resubmitting your figure files are available below the reviewer comments at the end of this letter.

If applicable, we recommend that you deposit your laboratory protocols in protocols.io to enhance the reproducibility of your results. Protocols.io assigns your protocol its own identifier (DOI) so that it can be cited independently in the future. For instructions see: https://journals.plos.org/plosone/s/submission-guidelines#loc-laboratory-protocols. Additionally, PLOS ONE offers an option for publishing peer-reviewed Lab Protocol articles, which describe protocols hosted on protocols.io. Read more information on sharing protocols at https://plos.org/protocols?utm_medium=editorial-email&utm_source=authorletters&utm_campaign=protocols.

As the corresponding author, your ORCID iD is verified in the submission system and will appear in the published article. PLOS supports the use of ORCID, and we encourage all coauthors to register for an ORCID iD and use it as well. Please encourage your coauthors to verify their ORCID iD within the submission system before final acceptance, as unverified ORCID iDs will not appear in the published article. *Only*  the individual author can complete the verification step; PLOS staff *cannot*  verify ORCID iDs on behalf of authors.

We look forward to receiving your revised manuscript.

Kind regards,

Tien Anh Tran

Academic Editor

PLOS One

Journal Requirements:

1. If the reviewer comments include a recommendation to cite specific previously published works, please review and evaluate these publications to determine whether they are relevant and should be cited. There is no requirement to cite these works unless the editor has indicated otherwise.

[Note: HTML markup is below. Please do not edit.]

Reviewer's Responses to Questions

**Comments to the Author**

Reviewer #1: (No Response)

**********

2. Is the manuscript technically sound, and do the data support the conclusions?

Reviewer #1: (No Response)

**********

3. Has the statistical analysis been performed appropriately and rigorously?

Reviewer #1: (No Response)

**********

4. Have the authors made all data underlying the findings in their manuscript fully available?

The PLOS Data policy

Reviewer #1: (No Response)

**********

5. Is the manuscript presented in an intelligible fashion and written in standard English?

Reviewer #1: (No Response)

**********

Reviewer #1: 1. Discuss deposit formation or fuel system compatibility associated with CNSL-containing biodiesel blends.

2. Authors should compare their BTE results with recent studies on ternary and non-edible biodiesel blends operating in conventional diesel engines LIKE "Optimization of Combustion, Performance, and Emission Characteristics of a Reactivity Controlled Compression Ignition Engine Fueled with Hemp Oil Methyl Ester and Pine Oil Blends using Response Surface Methodology"

3. The effect of biodiesel oxygen content on NOx formation requires a more discussion on thermal NOx rather than a description. "Comparative analysis of a diesel engine fueled with hydrogen-enriched nanoparticle-emulsified second-generation biodiesel"

4. Discuss cold-flow properties of the prepared blends.

**********

what does this mean?). If published, this will include your full peer review and any attached files.

If you choose “no”, your identity will remain anonymous but your review may still be made public.

**Do you want your identity to be public for this peer review?**  For information about this choice, including consent withdrawal, please see our Privacy Policy

Reviewer #1: No

**********

[NOTE: If reviewer comments were submitted as an attachment file, they will be attached to this email and accessible via the submission site. Please log into your account, locate the manuscript record, and check for the action link "View Attachments". If this link does not appear, there are no attachment files.]

To ensure your figures meet our technical requirements, please review our figure guidelines: https://journals.plos.org/plosone/s/figures

You may also use PLOS’s free figure tool, NAAS, to help you prepare publication quality figures: https://journals.plos.org/plosone/s/figures#loc-tools-for-figure-preparation.

NAAS will assess whether your figures meet our technical requirements by comparing each figure against our figure specifications.

# Author comment: Author response to Decision Letter 2

**DOI:** 10.1371/journal.pone.0360637.r004

21 Jul 2026

Detailed response is included in the response sheet enclosed with this revised submission.

Attachment Submitted filename: Response.docx

# Peer review: Decision Letter 2

**DOI:** 10.1371/journal.pone.0360637.r005

**Authors:** Tien Anh Tran

21 Aug 2026

Dear Dr. Dennison,

Thank you for submitting your manuscript to PLOS One. After careful consideration, we feel that it has merit but does not fully meet PLOS One’s publication criteria as it currently stands. Therefore, we invite you to submit a revised version of the manuscript that addresses the points raised during the review process.

==============================

==============================

Please submit your revised manuscript by Oct 05 2026 11:59PM. If you will need more time than this to complete your revisions, please reply to this message or contact the journal office at plosone@plos.org. When you're ready to submit your revision, log on to https://www.editorialmanager.com/pone/ and select the 'Submissions Needing Revision' folder to locate your manuscript file.

- A letter that responds to each point raised by the academic editor and reviewer(s). You should upload this letter as a separate file labeled 'Response to Reviewers'.
- A marked-up copy of your manuscript that highlights changes made to the original version. You should upload this as a separate file labeled 'Revised Manuscript with Track Changes'.
- An unmarked version of your revised paper without tracked changes. You should upload this as a separate file labeled 'Manuscript'.

If you would like to make changes to your financial disclosure, please include your updated statement in your cover letter. Guidelines for resubmitting your figure files are available below the reviewer comments at the end of this letter.

If applicable, we recommend that you deposit your laboratory protocols in protocols.io to enhance the reproducibility of your results. Protocols.io assigns your protocol its own identifier (DOI) so that it can be cited independently in the future. For instructions see: https://journals.plos.org/plosone/s/submission-guidelines#loc-laboratory-protocols. Additionally, PLOS One offers an option for publishing peer-reviewed Lab Protocol articles, which describe protocols hosted on protocols.io. Read more information on sharing protocols at https://plos.org/protocols?utm_medium=editorial-email&utm_source=authorletters&utm_campaign=protocols.

As the corresponding author, your ORCID iD is verified in the submission system and will appear in the published article. PLOS supports the use of ORCID, and we encourage all coauthors to register for an ORCID iD and use it as well. Please encourage your coauthors to verify their ORCID iD within the submission system before final acceptance, as unverified ORCID iDs will not appear in the published article. *Only*  the individual author can complete the verification step; PLOS staff *cannot*  verify ORCID iDs on behalf of authors.

We look forward to receiving your revised manuscript.

Kind regards,

Tien Anh Tran

Academic Editor

PLOS One

**Journal Requirements:**

1. If the reviewer comments include a recommendation to cite specific previously published works, please review and evaluate these publications to determine whether they are relevant and should be cited. There is no requirement to cite these works unless the editor has indicated otherwise.

2. Please review your reference list to ensure that it is complete and correct. If you have cited papers that have been retracted, please include the rationale for doing so in the manuscript text, or remove these references and replace them with relevant current references. Any changes to the reference list should be mentioned in the rebuttal letter that accompanies your revised manuscript. If you need to cite a retracted article, indicate the article’s retracted status in the References list and also include a citation and full reference for the retraction notice.

[Note: HTML markup is below. Please do not edit.]

Reviewers' comments:

Reviewer's Responses to Questions

**Comments to the Author**

Reviewer #1: All comments have been addressed

Reviewer #3: (No Response)

**********

2. Is the manuscript technically sound, and do the data support the conclusions?

Reviewer #1: Yes

Reviewer #3: Yes

**********

3. Has the statistical analysis been performed appropriately and rigorously?

Reviewer #1: N/A

Reviewer #3: Yes

**********

4. Have the authors made all data underlying the findings in their manuscript fully available?

The PLOS Data policy

Reviewer #1: Yes

Reviewer #3: Yes

**********

5. Is the manuscript presented in an intelligible fashion and written in standard English?

Reviewer #1: Yes

Reviewer #3: Yes

**********

Reviewer #1: After revision, the authors have satisfactorily addressed all reviewer comments. The manuscript is now suitable for publication.

Reviewer #3: The authors have made generally reasonable revisions in response to the previous review comments, particularly by expanding the discussions on fuel-system compatibility and deposit formation, comparison of brake thermal efficiency with recent studies, thermal NOx formation mechanisms, and cold-flow characteristics. These revisions have improved the manuscript and addressed several of the concerns raised in the previous round of review. Nevertheless, several key issues related to data consistency overall structural coherence remain unresolved. These issues should be satisfactorily addressed before the manuscript can be reconsidered for publication:

(1) The authors have added a new Section 3.2.5 as a comparison for B20. However, this B20 literature comparison is placed under Section 3.2 "B10 Performance Characteristics," where the actual B20 results have not yet been presented. In addition, the heading of the immediately following Section 3.3 is incorrectly given as "B10," whereas its content in fact concerns B20. Please correct the section arrangement and headings accordingly.

(2) The text states that the NOx emissions of both B20 and B30 are higher than those of diesel. In Fig. 15, diesel and B10 are both approximately 962 ppm, B20 approximately 1069 ppm, and B30 approximately 1019 ppm. These values do not correspond to those reported in the Conclusions.

(3) The fuel blends given in the text are only B10, B20 and B30, and the engine test section explicitly states that the test fuels were diesel, B10, B20 and B30. Nevertheless, the Conclusions section presents test results corresponding to B40.

(4) Please check for citation mismatches. For example, the text cites Ref. [16] in support of the "performance and emissions of waste frying oil methyl ester in a diesel engine at a higher compression ratio," whereas Ref. [16] is in fact a study on the co-pyrolysis kinetics of waste tyre and walnut shell.

(5) Some of the BSFC values reported in the paper are inconsistent with the TFC and BP values given in the tables. The authors should further verify the relevant calculations.

**********

what does this mean?). If published, this will include your full peer review and any attached files.

If you choose “no”, your identity will remain anonymous but your review may still be made public.

**Do you want your identity to be public for this peer review?**  For information about this choice, including consent withdrawal, please see our Privacy Policy

Reviewer #1: No

Reviewer #3: No

**********

[NOTE: If reviewer comments were submitted as an attachment file, they will be attached to this email and accessible via the submission site. Please log into your account, locate the manuscript record, and check for the action link "View Attachments". If this link does not appear, there are no attachment files.]

To ensure your figures meet our technical requirements, please review our figure guidelines: https://journals.plos.org/plosone/s/figures

You may also use PLOS’s free figure tool, NAAS, to help you prepare publication quality figures: https://journals.plos.org/plosone/s/figures#loc-tools-for-figure-preparation.

NAAS will assess whether your figures meet our technical requirements by comparing each figure against our figure specifications.

# Author comment: Author response to Decision Letter 3

**DOI:** 10.1371/journal.pone.0360637.r006

8 Sep 2026

Review Comments to the Author

Please use the space provided to explain your answers to the questions above. You may also include additional comments for the author, including concerns about dual publication, research ethics, or publication ethics. (Please upload your review as an attachment if it exceeds 20,000 characters)

Reviewer #1: After revision, the authors have satisfactorily addressed all reviewer comments. The manuscript is now suitable for publication.

We thank the reviewer for the recommendation.

Reviewer #3: The authors have made generally reasonable revisions in response to the previous review comments, particularly by expanding the discussions on fuel-system compatibility and deposit formation, comparison of brake thermal efficiency with recent studies, thermal NOx formation mechanisms, and cold-flow characteristics. These revisions have improved the manuscript and addressed several of the concerns raised in the previous round of review. Nevertheless, several key issues related to data consistency overall structural coherence remain unresolved. These issues should be satisfactorily addressed before the manuscript can be reconsidered for publication:

1. The authors have added a new Section 3.2.5 as a comparison for B20. However, this B20 literature comparison is placed under Section 3.2 "B10 Performance Characteristics," where the actual B20 results have not yet been presented. In addition, the heading of the immediately following Section 3.3 is incorrectly given as "B10," whereas its content in fact concerns B20. Please correct the section arrangement and headings accordingly.

We thank the reviewer for pointing out this inconsistency in our organization. We have carefully reorganized sections 3.2 and 3.3 so that the explanation of each biodiesel mix is placed in the correct section. The header of Section 3.3 has been changed from the wrong one to “Performance Characteristics of B20 Biofuel Blend” and the B20 literature comparison has been relocated to the B20 subsection after presenting and discussing the B20 experimental results. The numbering of the respective sub-paragraphs has been revised to bring uniformity in the work. The heading has been updated under the section 3.3 as 3.3.5.

2. The text states that the NOx emissions of both B20 and B30 are higher than those of diesel. In Fig. 15, diesel and B10 are both approximately 962 ppm, B20 approximately 1069 ppm, and B30 approximately 1019 ppm. These values do not correspond to those reported in the Conclusions.

We thank the reviewer for the discrepancy. We have revised the discussion and conclusions in light of our re-evaluation of the NOx emissions data shown in Figure 15. The corrected average NOx emissions for the blends were measured at 962 ppm (B10), 1069 ppm (B20), and 1019 ppm (B30), while neat diesel produced 1092 ppm. So B30 is less than diesel but more than B10 and B20 is more than diesel. In this way, the mistake on the trend of NOx has been corrected.

This has bee updated under the section 3.5.2 and in the conclusion

3. The fuel blends given in the text are only B10, B20 and B30, and the engine test section explicitly states that the test fuels were diesel, B10, B20 and B30. Nevertheless, the Conclusions section presents test results corresponding to B40.

We thank the reviewer for this comment. The reference to B40 in the Conclusions was a typographical error, as B40 is not an experimentally studied fuel blend. Following the experimental approach, the inquiry was limited to neat diesel, B10, B20 and B30 mixes only. The incorrect reference to B40 has been removed from the Conclusions and the sentence has been changed accordingly so that it will keep consistent with the experimental scope of the study.

4. Please check for citation mismatches. For example, the text cites Ref. [16] in support of the "performance and emissions of waste frying oil methyl ester in a diesel engine at a higher compression ratio," whereas Ref. [16] is in fact a study on the co-pyrolysis kinetics of waste tyre and walnut shell.

We thank the reviewer for this valuable suggestion. Reference list and citations in the manuscript were reviewed. We checked Ref. [16] which is a research paper on thermogravimetric properties and co-pyrolysis kinetics of scrap tire and walnut shell and it did not support the claim of waste frying oil methyl ester and increased compression ratio. So, we have taken out the unfounded and misattributed statement. We also looked at surrounding references to confirm that the literature cited supports the claims it is used for.

The reference [16] is a study of co-pyrolysis, not a study of biodiesel engines as listed in the references. This statement is not supported by the given Ref. [16] It is left out altogether.

5. Some of the BSFC values reported in the paper are inconsistent with the TFC and BP values given in the tables. The authors should further verify the relevant calculations.

The BSFC values reported in the manuscript were carefully rechecked using the corresponding total fuel consumption (TFC) and brake power (BP) values given in the experimental tables. The corresponding figures and discussions have also been reviewed and updated to ensure full consistency between TFC, BP and BSFC throughout the revised manuscript. We would like to thank the reviewer for the help in improving the accuracy and consistency of the reported results.

Attachment Submitted filename: Comment.docx

# Peer review: Decision Letter 3

**DOI:** 10.1371/journal.pone.0360637.r007

**Authors:** Tien Anh Tran

29 Sep 2026

Performance and Emission Analysis of Sustainable Rice Bran-Calophyllum-CNSL Biodiesel Blends in a Conventional Diesel Engine

PONE-D-26-08657R3

Dear Dr. Dennison,

We’re pleased to inform you that your manuscript has been judged scientifically suitable for publication and will be formally accepted for publication once it meets all outstanding technical requirements.

Within one week, you’ll receive an e-mail detailing the required amendments. When these have been addressed, you’ll receive a formal acceptance letter and your manuscript will be scheduled for publication.

An invoice will be generated when your article is formally accepted. Please note, if your institution has a publishing partnership with PLOS and your article meets the relevant criteria, all or part of your publication costs will be covered. Please make sure your user information is up-to-date by logging into Editorial Manager at Editorial Manager® and clicking the ‘Update My Information' link at the top of the page. For questions related to billing, please contact billing support.

If your institution or institutions have a press office, please notify them about your upcoming paper to help maximize its impact. If they’ll be preparing press materials, please inform our press team as soon as possible -- no later than 48 hours after receiving the formal acceptance. Your manuscript will remain under strict press embargo until 2 pm Eastern Time on the date of publication. For more information, please contact onepress@plos.org.

Kind regards,

Tien Anh Tran

Academic Editor

PLOS One

Additional Editor Comments (optional):

Reviewers' comments:

Reviewer's Responses to Questions

**Comments to the Author**

Reviewer #1: All comments have been addressed

**********

2. Is the manuscript technically sound, and do the data support the conclusions?

Reviewer #1: Yes

**********

3. Has the statistical analysis been performed appropriately and rigorously?

Reviewer #1: Yes

**********

4. Have the authors made all data underlying the findings in their manuscript fully available?

The PLOS Data policy

Reviewer #1: Yes

**********

5. Is the manuscript presented in an intelligible fashion and written in standard English?

Reviewer #1: Yes

**********

Reviewer #1: As per the comments raised during the previous review round, the authors have carefully considered and addressed all the reviewer comments. The necessary revisions have been incorporated in the revised manuscript.

**********

what does this mean?). If published, this will include your full peer review and any attached files.

If you choose “no”, your identity will remain anonymous but your review may still be made public.

**Do you want your identity to be public for this peer review?**  For information about this choice, including consent withdrawal, please see our Privacy Policy

Reviewer #1: No

**********

# Editor report: Acceptance letter

**DOI:** 10.1371/journal.pone.0360637.r008

**Authors:** Tien Anh Tran

PONE-D-26-08657R3

PLOS One

Dear Dr. Dennison,

I'm pleased to inform you that your manuscript has been deemed suitable for publication in PLOS One. Congratulations! Your manuscript is now being handed over to our production team.

At this stage, our production department will prepare your paper for publication. This includes ensuring the following:

* All references, tables, and figures are properly cited

* All relevant supporting information is included in the manuscript submission,

* There are no issues that prevent the paper from being properly typeset

You will receive further instructions from the production team, including instructions on how to review your proof when it is ready. Please keep in mind that we are working through a large volume of accepted articles, so please give us a few days to review your paper and let you know the next and final steps.

Lastly, if your institution or institutions have a press office, please let them know about your upcoming paper now to help maximize its impact. If they'll be preparing press materials, please inform our press team within the next 48 hours. Your manuscript will remain under strict press embargo until 2 pm Eastern Time on the date of publication. For more information, please contact onepress@plos.org.

You will receive an invoice from PLOS for your publication fee after your manuscript has reached the completed accept phase. If you receive an email requesting payment before acceptance or for any other service, this may be a phishing scheme. Learn how to identify phishing emails and protect your accounts at https://explore.plos.org/phishing.

If we can help with anything else, please email us at customercare@plos.org.

Thank you for submitting your work to PLOS One and supporting open access.

Kind regards,

PLOS One Editorial Office Staff

on behalf of

Professor Tien Anh Tran

Academic Editor

PLOS One
